# A blended learning approach for teaching thoracic radiology to medical students: a proof-of-concept study

**DOI:** 10.3389/fmed.2023.1272893

**Published:** 2023-11-23

**Authors:** Fabian Stoehr, Yang Yang, Lukas Müller, Phyllis Gerstenmeier, Daniel Pinto dos Santos, Pavel Dietz, Andreas Weimer, Michael Ludwig, Roman Kloeckner, Johannes Matthias Weimer

**Affiliations:** ^1^Department of Diagnostic and Interventional Radiology, University Medical Center of the Johannes Gutenberg-University Mainz, Mainz, Germany; ^2^Institute of Diagnostic and Interventional Radiology, University Hospital Frankfurt, Frankfurt, Germany; ^3^Department of Diagnostic and Interventional Radiology, University Hospital of Cologne, Cologne, Germany; ^4^Institute of Occupational, Social, and Environmental Medicine, University Medical Center of the Johannes Gutenberg-University Mainz, Mainz, Germany; ^5^Center of Orthopedics, Trauma Surgery, and Spinal Cord Injury, Heidelberg Trauma Research Group, Heidelberg University Hospital, Heidelberg, Germany; ^6^Department of Internal Medicine I, Hospital of the German Armed Forces Berlin, Berlin, Germany; ^7^Institute of Interventional Radiology, University Hospital Schleswig-Holstein – Campus Lübeck, Lübeck, Germany; ^8^Rudolf Frey Lernklinik, University Medical Center of the Johannes Gutenberg-University Mainz, Mainz, Germany

**Keywords:** medical education, online learning, blended learning, radiology education, thoracic imaging

## Abstract

**Introduction:**

The best way to impart knowledge to medical students is still unclear. Therefore, we designed a blended learning course in thoracic radiology including both “traditional” in-class time as well as online learning modules. The aims were (1) to investigate students’ attitudes toward this blended learning approach; and (2) to test whether it improved their knowledge about thoracic radiology.

**Methods:**

A prospective study was conducted at the local medical center; 156 fourth-year medical students completed this study. Before and after the course, students had to complete (1) questionnaires to investigate their attitudes (7-point Likert scale); and (2) an objective test to assess their knowledge (multiple-choice/free text questions; results as % of correct answers).

**Results:**

Regarding (1), the course led to an improvement in all items compared to baseline, exemplary: interest in thoracic radiology (precourse 4.2 vs. 5.4 postcourse) and the fulfillment of students’ expressed requirements regarding the teaching content (4.5 precourse vs. 6.2 postcourse). Furthermore, the great majority (88%) of our participants wished for more online learning offerings in the future. Regarding (2), the course led to improved knowledge on the objective test (precourse: 40% vs. postcourse: 63% correct answers).

**Conclusion:**

This feasibility study showed the successful design and implementation of a blended learning approach in thoracic radiology. Furthermore, it revealed medical students’ positive attitudes toward this approach and showed an increased knowledge in thoracic radiology. Thus, such approaches might be used to enrich the teaching armamentarium in medical education and to further enhance interest and knowledge in thoracic diseases among medical students.

## 1 Introduction

The best way to impart knowledge to medical students is still unclear and remains the focus of ongoing research (1–4). In the vast majority of medical schools, teaching is still based mainly on traditional, *ex cathedra* concepts (4–6). Previous studies have proposed that students’ discontent with the current teaching system might be one of the various reasons for their declining participation rates in recent years (2, 7–9).

However, there is growing interest in more innovative, student-centered approaches including, in particular, online teaching and learning (2, 9, 10). As a reaction, such online-based formats have been added to radiology education curricula, with great success regarding the effectiveness of learning and positively influencing students’ attitudes toward learning (11–16). Notably, blended learning approaches attracted much attention in recent years (17–20). In its essence, blended learning combines both online and onsite learning resulting in a beneficial mix and allowing for a possible greater educational impact (19–21). In a “flipped classroom,” an autonomous, online-based learning phase precedes a face-to-face, onsite learning session (18, 22). In the online phase, students actively and autonomously acquire knowledge serving as a basis for the face-to-face phase. In the subsequent onsite phase, the main focus is to deepen the acquired knowledge and to further enhance the learning process (18, 20).

If used properly, this shift from instructor-centered teaching to student-centered learning can promote accelerated learning and might foster i.a. the learning and motivation (18–21).

On the other hand, the success of this blended learning course strongly depends on an adequate acquisition of knowledge in the online phase (18, 22). Thus, students’ motivation to actively participate and to prepare themselves prior to the face-to-face session is crucial. From lecturers’ perspective, a “traditional” onsite course has to be transformed into an online/hybrid format (14, 18). As a dedicated teaching platform and multimedia material is required, this requires additional effort and can be a time-consuming and potentially expensive process.

Over the last 2 years, the coronavirus disease 2019 (COVID-19) pandemic has acted as a catalyst for such innovative concepts as lecturers were forced to critically rethink teaching formats in medical education (22–28). As a positive consequence, several attempts have been made to implement various teaching formats (22–26). However, as courses were mostly implemented under considerable time pressure, they can be seen as “emergency remote teaching” instead of dedicated and embedded in a structured, curricular framework. Nevertheless, even after overcoming the pandemic, such innovative and “digitally supported” teaching concepts are not provided throughout all medical universities.

Thus, we designed a blended learning course in thoracic radiology and implemented it into the teaching curriculum of our faculty. In the scenario chosen for this study and as mentioned above, students had to acquire basic knowledge independently outside the classroom before attending a live didactic course (17, 29). For this purpose, we developed an online platform, which served as a basis for the online education part.

We deliberately chose thoracic radiology as the subject because it is (1) a common field in diagnostic imaging; and (2) includes basic knowledge almost every medical student will need as a doctor (30, 31). First, we aimed to investigate students’ general interest in this blended learning approach. Second, we aimed to test whether this specific course design can improve students’ knowledge and understanding about thoracic radiology.

## 2 Methods

### 2.1 Study setup and course design

This study was conducted prospectively at the Department of Diagnostic and Interventional Radiology, University Medical Center Mainz. It was performed in accordance with the “Checklist for Reporting Results of Internet E-Surveys (CHERRIES)” (32) and with the “Strengthening the Reporting of Observational Studies in Epidemiology (STROBE) statement: guidelines for reporting observational studies” (33) (Checklist S1). Institutional review board approval was waived by the Ethics Committee of the Medical Association of Rhineland-Palatinate.

**Figure 1:**
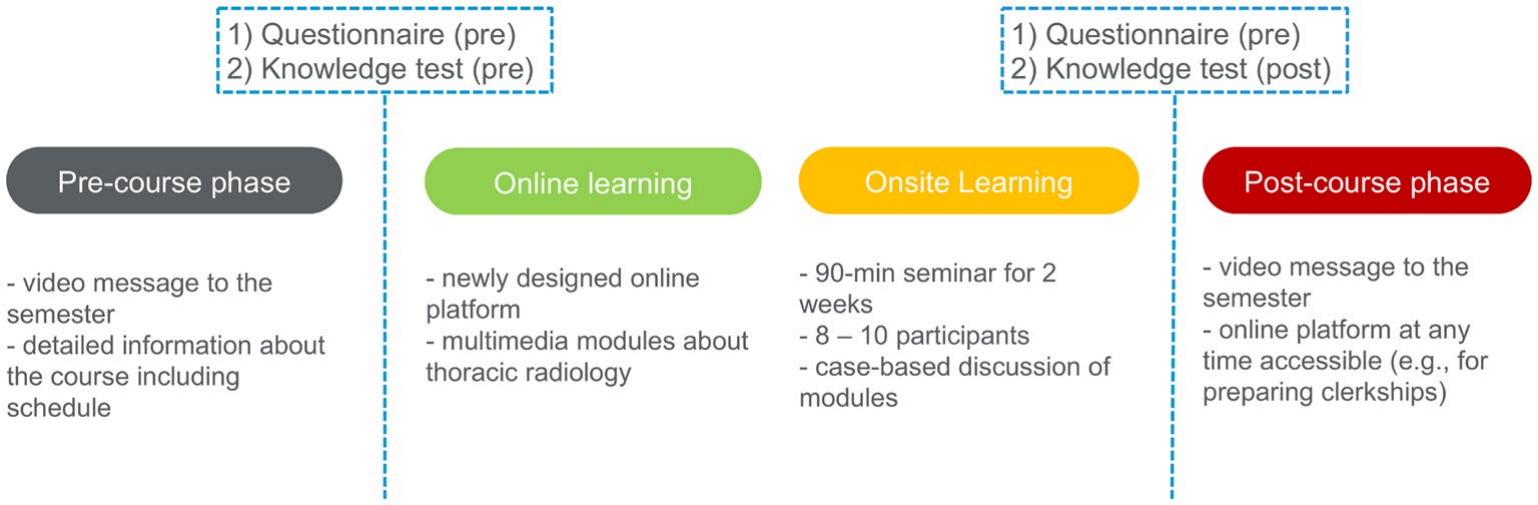
Overview of the setup of our blended learning course in thoracic radiology.

For this study, a dedicated course on thoracic radiology was designed using a blended learning approach. The course was part of the officially predetermined curriculum of our university medical center, delivered to fourth-year medical students and embedded into a pre-existing curricular course in radiology; other courses of the officially predetermined curriculum included i.a. Interventional Radiology, Cardiovascular Radiology, Oncological Imaging and Neuroradiology. It was conducted as a hybrid course consisting of a structured online learning platform and one 90-min dedicated onsite course with face-to-face teaching. Before the online part as well as after the onsite part, participants’ knowledge about thoracic radiology as well as their attitudes toward the course design was captured. To this end, dedicated questionnaires as well as an objective knowledge test were designed (see section 2.3 for more details). Before each part of the course (online and onsite), participants were informed that the survey results would be anonymous and that they were collected for research purposes only. Figure 1 gives an overview of the setup of our blended learning course.

### 2.2 Course program (online and onsite)

The course program was based on the “Radiological Curriculum for Undergraduate Medical Education in Germany” of the German Radiological Society (DRG) (30) as well as on the “Curriculum for Undergraduate Radiological Education” of the European Society of Radiology (ESR) (31). Design and implementation of the course followed the waterfall model (34) and was a multi-stage process.

Online teaching was performed via a dedicated, newly designed online learning platform. Content was composed by a consortium of several experts in the field of thoracic radiology, pulmonology, and didactics. Images were drawn by a web designer; the online learning platform was built by a professional programmer and could be accessed via a standard web browser. The platform consisted of six modules, which had to be studied by the students autonomously before the onsite course was given. Modules transmitted knowledge about technical basics as well as normal and pathological findings in X-ray, CT, and ultrasonography. Modules were: basics chest X-ray, basics chest CT, basics ultrasonography, pathological findings chest imaging, pathological findings CT/X-ray and pathological findings ultrasonography. An example of the structure of the online learning platform is provided in the Supplementary Figure S1.

In order to enhance the learning experience, online content was enriched with different features (clickable color highlighting, video loops on demand, image magnification). An example of how each of these features was used in the platform is provided the Supplementary Figure S2.

Onsite courses were given as 90-min face-to-face seminars. Based on the modules mentioned above, there was a case-based discussion in which the participants could deepen their understanding in thoracic radiology. In order to have the same knowledge base, modules of the onsite and online part matched. Courses were carried out as block training on a daily basis during 2 weeks in July 2022 and given by consultant radiologists with considerable experience in thoracic imaging. Group size for the onsite courses was 8–10 students.

### 2.3 Questionnaire and test design

A dedicated questionnaire was designed together with the Institute of Occupational, Social and Environmental Medicine (ASU) of the University Medical Center Mainz. The questionnaire comprised various sections covering in particular the following topics: interest and knowledge of thoracic radiology; the students’ expressed requirements for teaching content and teaching medium in thoracic radiology; the use of online learning in general and in thoracic radiology; attitudes toward online learning before, during, and after the pandemic; and technical aspects of online learning as well as the current and possible future role of online learning. In the questionnaire, particular attention was paid to the assessment of the online learning platform as it was newly designed for this course. Participants were asked to answer the questions using a 7-point Likert scale. Compared with a 5-point scaling system, a 7-point Likert scale provides higher variance and thus higher reliability. Other scales such as 9- or even 11-point scales would not add any more value regarding the information obtained and could even strain our participants’ abstraction capabilities. The questionnaires are provided in the Supplementary material (Precourse evaluation S2 and Postcourse evaluation S3).

The objective test was based on the content of this thoracic radiology course as well as on the curricula of the “German Radiological Society” (DRG) and the “European Society of Radiology” (ESR) for undergraduate radiological teaching (30, 31). It was composed by a consortium of several experts in the field of thoracic radiology, pulmonology, and didactics. For a more balanced assessment, multiple-choice as well as free text questions were used. Design of the questions corresponded to current specifications (35, 36). The entire test is provided in the Supplementary material (Quiz thoracic radiology S4).

### 2.4 Validation

The questionnaires and objective test underwent a two-step validation to further enhance the quality of the study. First, cognitive pretesting was performed on a sample of 10 participants (medical students in their last year; feedback was given via oral survey) (37); second, pilot testing was performed on a cohort of 25 participants (medical students in their last year; feedback was given via written survey) (38).

### 2.5 Data collection and statistical analysis

Survey results were collected via an established online survey tool (SurveyMonkey^1^). After the questionnaires and the test were designed, web links were encoded in QR codes, which were implemented in the presentations of the courses. Students were asked to scan the QR codes with their smartphones to access the questionnaires. In case of technical problems, we provided tablets. Final survey results were exported from SurveyMonkey as a CSV file and subsequently analyzed using R 4.2.2 (A Language and Environment for Statistical Computing, R Foundation for Statistical Computing^2^; last accessed February 2023). Figures were plotted using the ggplot2 and Likert packages (39). Means and standard deviations were calculated to analyze the results regarding descriptive statistical analysis (40), regression analysis was performed to identify possible relationships between variables (see section 3.3 for detailed description).

## 3 Results

### 3.1 Participants’ demographics

In total, 156 of 160 students completed the questionnaires (97.5% response rate). Demographic data recorded were gender (40.4% male, 59.6% female), age (mean 25.9 years), current study year (mean fourth study year) as well as possible vocational training before medical studies. A tabular overview is provided in the Supplementary Table S5.

### 3.2 Survey results

**Figure 2:**
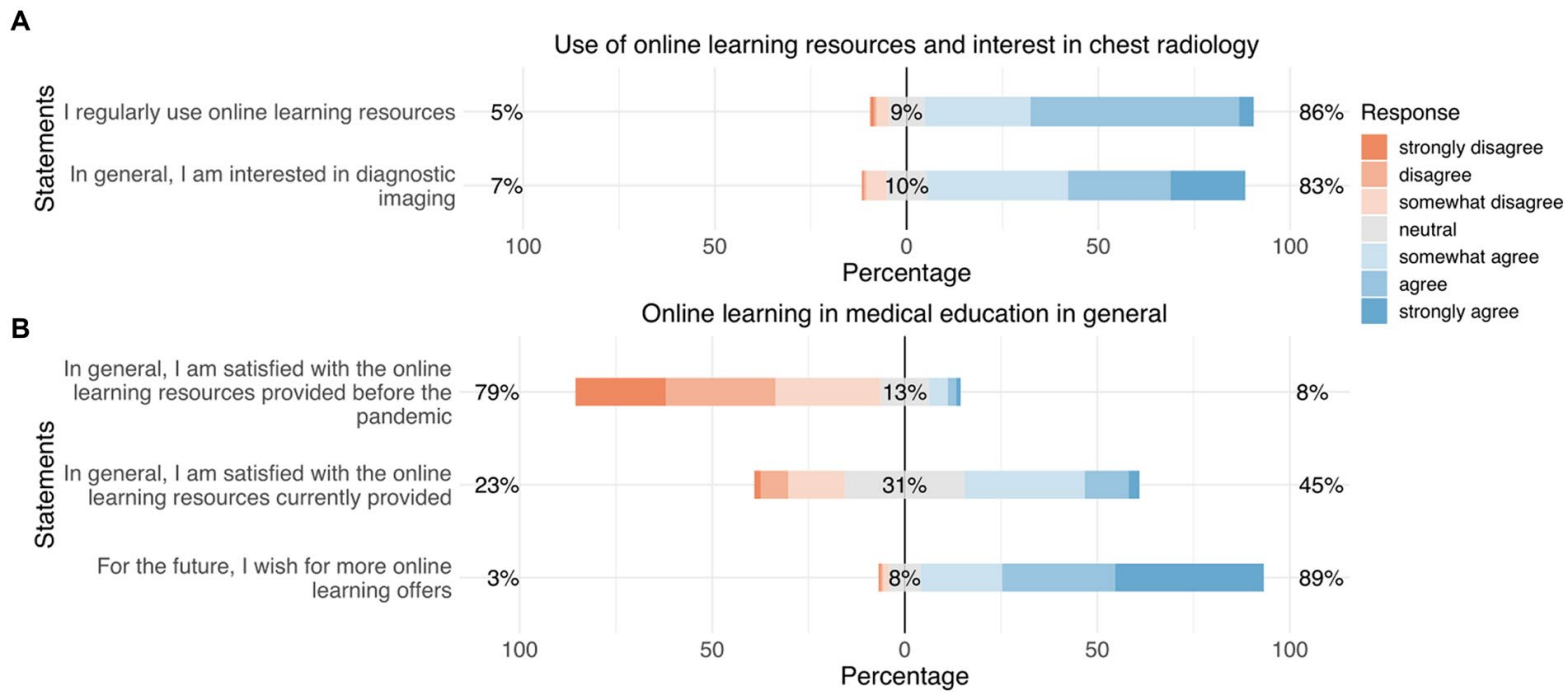
Responses regarding “use of online learning resources and interest in thoracic radiology” **(A)** and “online learning in medical education in general” **(B)**. Orange represents “disagreement,” gray represents “neutral,” and blue represents “agreement.”

**Figure 3:**
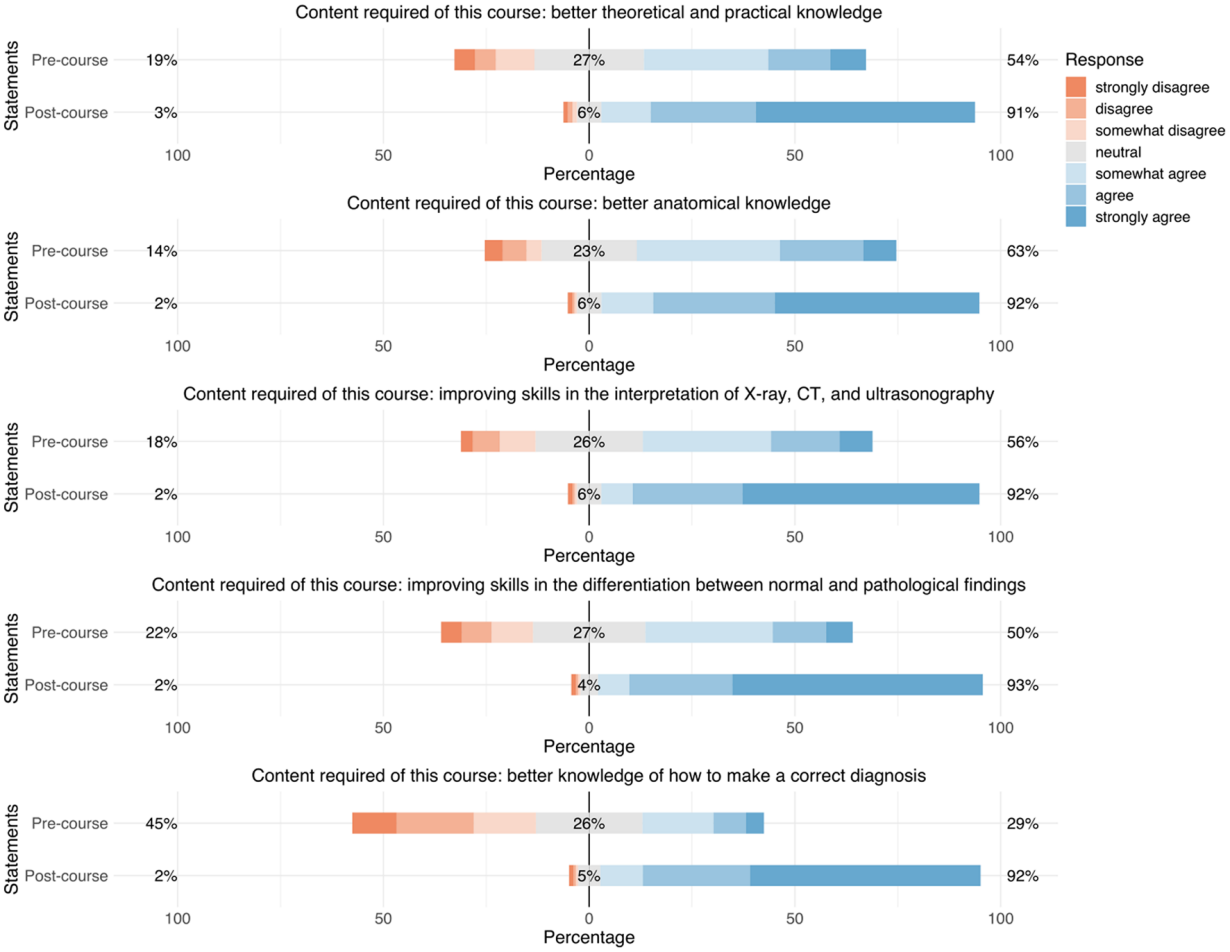
Responses regarding “requirements on teaching content in thoracic radiology.” Precourse results are compared to postcourse results. Orange represents “disagreement,” gray represents “neutral,” and blue represents “agreement.”

**Figure 4:**
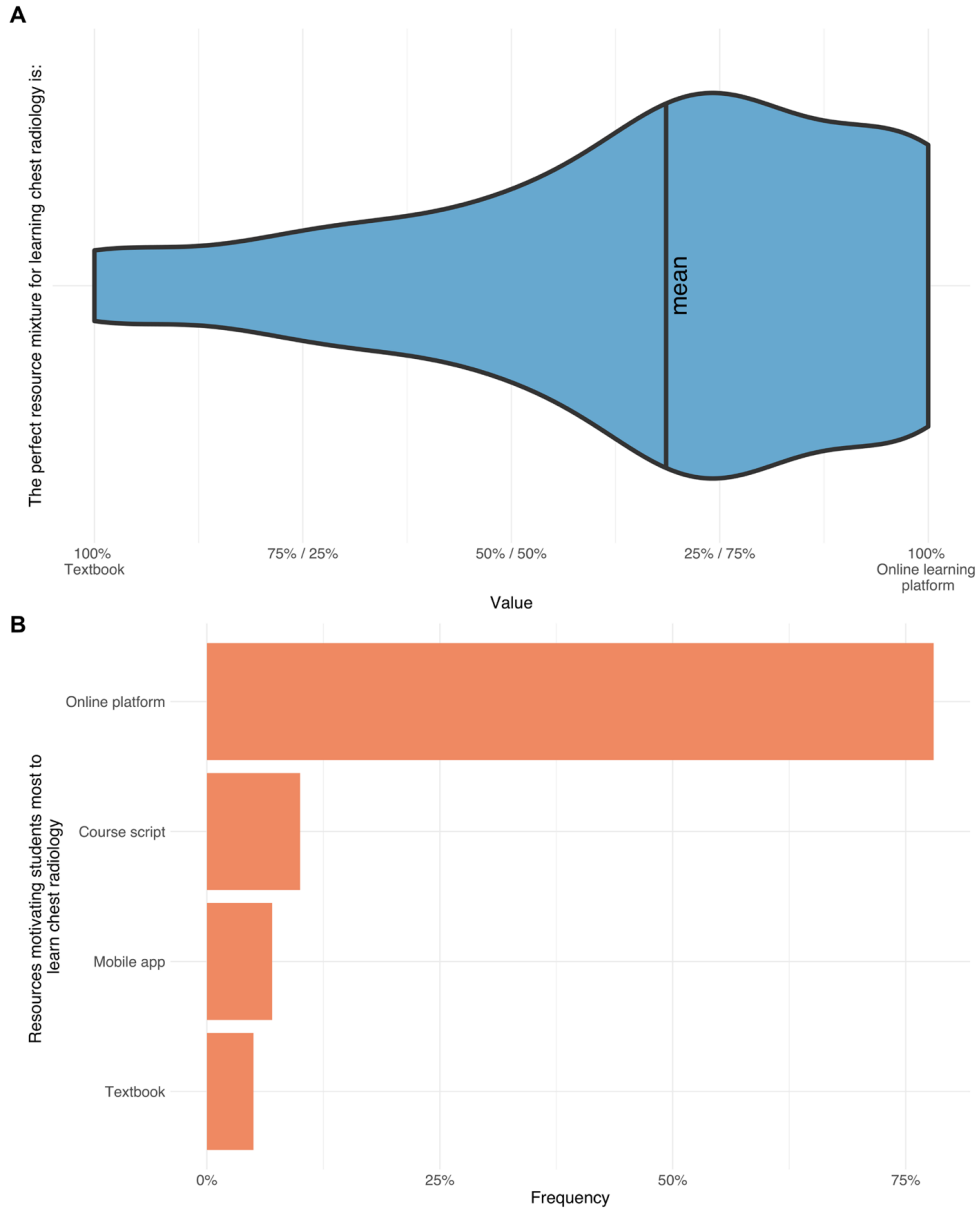
The upper violin plot depicts the preferred teaching type (online platform vs. textbook/lecture notes) (66%) **(A)**. The bars demonstrate the ranking of the teaching types motivating the participants most to learn thoracic radiology: Online platform (78%), course script (10%), app (7%), and textbook (5%) **(B)**.

**Figure 5:**
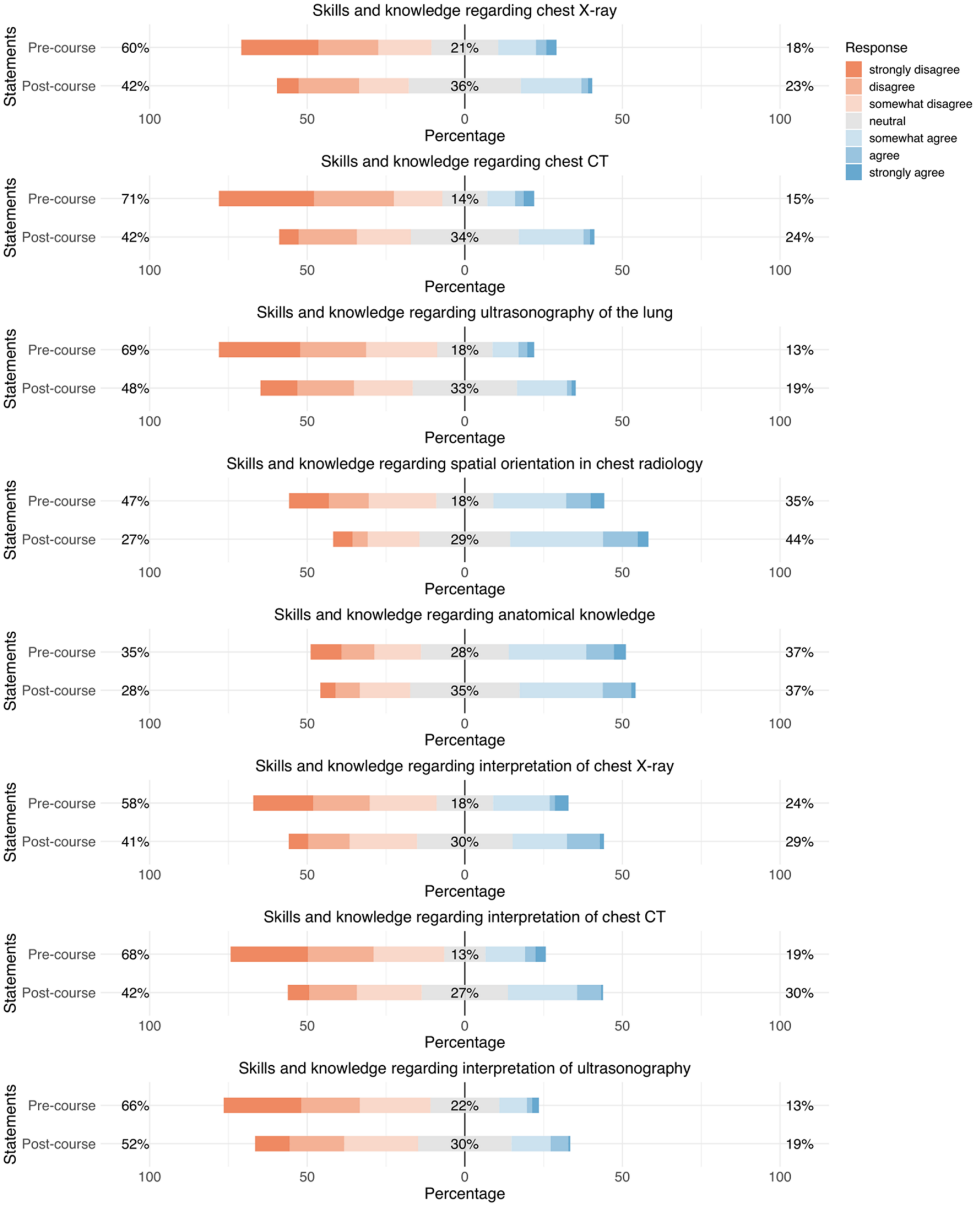
Responses regarding “subjective self-assessment of knowledge in thoracic radiology.” Precourse results are compared to postcourse results. Orange represents “disagreement,” gray represents “neutral,” and blue represents “agreement.”

**Figure 6:**
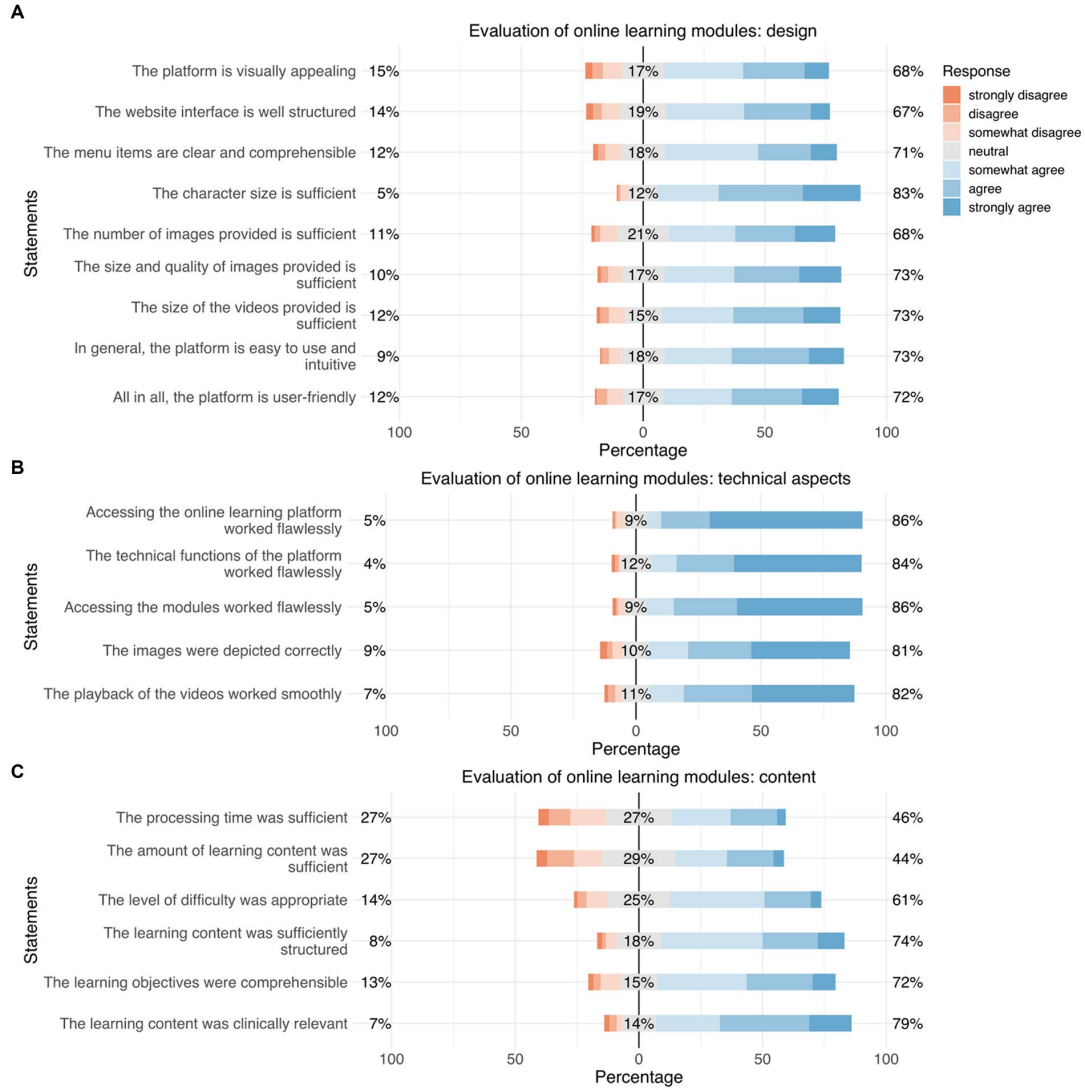
Responses regarding “evaluation of the online learning modules: design” **(A)**, “evaluation of the online learning modules: technical aspects” **(B)**, and “evaluation of the online learning modules: content” **(C)**. Orange represents “disagreement,” gray represents “neutral,” and blue represents “agreement.”

**Figure 7:**
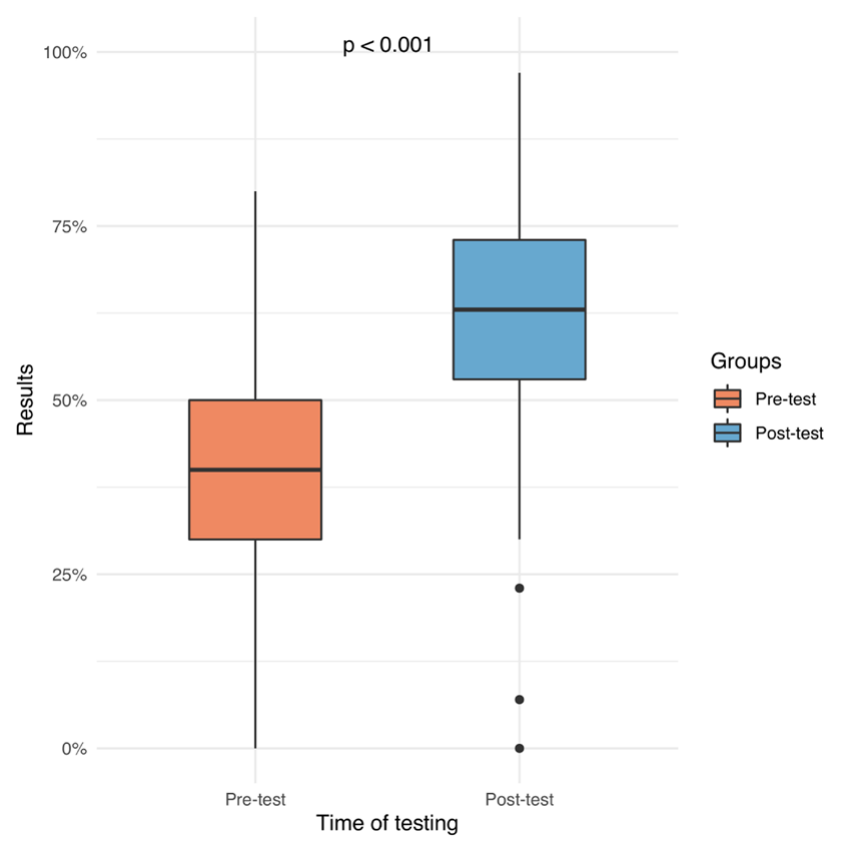
The left plot depicts the precourse results (mean 40%, IQR 30–50%). The right plot depicts the postcourse results (mean 63%, ICR 53–73%). This was statistically significant (*p* < 0.001).

**Figure 8:**
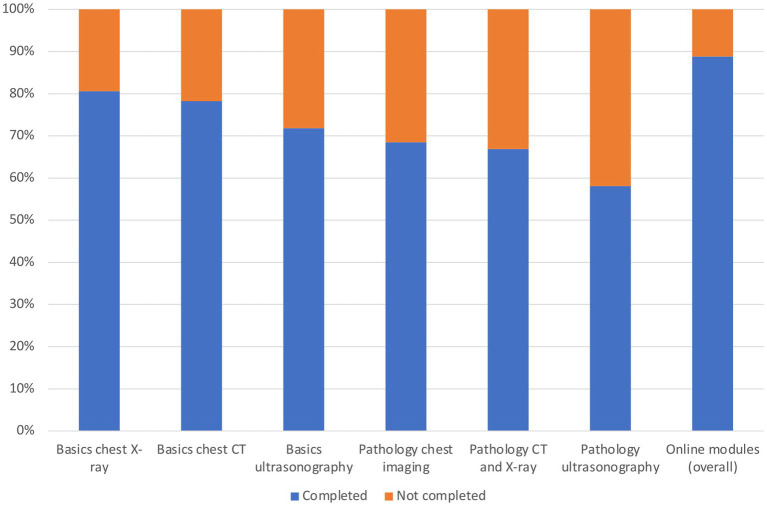
Completion rates of each module in %. Blue represents completion, whereas orange represents lack of completion/no completion.

The following survey results are presented in written form as well as graphically (Figures 2–8). To increase comprehensibility, the text contains only the key findings and summarizes “strongly disagree,” “disagree,” and “somewhat disagree” as disagreement, and “somewhat agree,” “agree,” and “strongly agree” as agreement. For statistical analyses, the original 7-point categories were used.

#### 3.2.1 Use of online learning resources and interest in thoracic radiology

A great majority of the participants stated that they use online learning media (86%), with an average of 12 h of use per week. This remained unchanged after the course. One-fourth of our participants had already used online learning media before (27%). If online learning media were used, online learning platforms were ranked in first place (16%), before eBooks (10%) and apps (12%).

Prior to the course, an overwhelming majority stated that they were interested in diagnostic imaging in thoracic radiology (83%). Before the course, only one-third of the participants stated that they had previous experience in thoracic radiology (31%), mostly from textbooks (37%), followed by curricular courses (21%) and webinars (11%) (Figure 2).

#### 3.2.2. Attitudes toward online learning in medical education in general

Only a few participants were satisfied with the online learning courses provided before the pandemic (8%). About the half of our participants were satisfied with the online courses provided during the pandemic (45%). However, the great majority of the participants wished for more online learning offerings in the future (89%) (Figure 2).

#### 3.2.3 Student demands for teaching content in thoracic radiology

Before the course, participants demanded a better theoretical and practical knowledge of thoracic radiology (54%), a better understanding of thoracic anatomy (63%), and to be able to better differentiate between pathological and normal findings (50%). After the course, an overwhelming majority of our participants stated that their requirements for teaching content were fulfilled regarding their theoretical and practical knowledge of thoracic radiology (91%), their understanding of thoracic anatomy (92%), and their ability to differentiate between pathological and normal findings (93%) (Figure 3).

#### 3.2.4 Students’ preferences and requirements for teaching media in thoracic radiology

Before the course, a great majority of the participants ranked online learning platforms as their preferred learning type that would motivate them the most to learn thoracic radiology (70%), before scripts/lecture notes (10%), apps (7%), and books (5%). After the course, these high expectations were fulfilled as the great majority stated that online learning platforms motivated them to become involved with thoracic radiology, even in the future (73%). Furthermore, students chose the online platform as their preferred teaching type (Figure 4).

#### 3.2.5 Subjective self-assessment of knowledge in thoracic radiology

Before the course, participants stated that they had rather little knowledge of thoracic radiology in general; for example, knowledge about X-ray (18%), CT (15%), ultrasonography (13%), spatial orientation (35%), and thoracic anatomy in general (37%) (Figure 5).

After the course, participants stated that they had the same or improved knowledge regarding all items; for example, knowledge about X-ray (59%), CT (58%), ultrasonography (52%), spatial orientation (73%), and thoracic anatomy in general (72%) (Figure 5).

#### 3.2.6 Evaluation of online learning modules regarding design, technical aspects, and content

In general, participants were satisfied with the online learning modules; for example, regarding user friendliness (72%), playback of the videos (82%), content (74%), and the level of difficulty of the learning materials (61%) (Figure 6).

### 3.3 Objective test before and after the course

Before the course, participants achieved a mean score of 40% on the objective test. After the course, participants achieved a mean score of 63% on the objective test (Figure 7).

Furthermore, a detailed analysis of the completion rates of the modules of our blended learning course was performed - broken down by each module separately. Results were depicted graphically (Figure 7) as well as in tabular form (Supplementary Table S7) (completion “yes/no” in %). Interestingly, a total of 11% of our participants did not complete any module.

Based on these results, we aimed to investigate if there is a possible relationship between the completion of the modules and the results in the knowledge test. For this purpose, (sub-) results of the knowledge test were analyzed, again broken down by each module separately. Supplementary Table S10 provides detailed information including results in pre- and posttest (mean ± standard deviation) and possible differences between pre- and posttest (“gain/loss of knowledge”) including its statistical significance (*p*-value) and its effect size (Cohen’s *d*). As a conclusion, regarding all modules there is a statistically significant knowledge gain between pre- and posttest.

In order to identify possible relationships between the completion of the modules and the (sub-) results in the posttest, a regression analysis was performed. Supplementary Table S8 provides a summary of the most important findings regarding the effect on the posttest. All data from the regression analysis (including analysis of the pretest) is provided in the Supplementary material (Supplementary Tables S8, S9).

In summary, completion of the modules leads to significantly better results in the posttest. However, even more interesting is the “no module” subgroup – those 11% of participants who did not complete any module (Supplementary Table S7). As those 11% only attended the onsite part of our blended learning course, they could somehow serve as a comparative internal reference group. According to our results, this subgroup has significantly worse results in the posttest regarding all modules (Supplementary Table S8). Of course, this conclusion must be considered with the utmost care as the “no module” subgroup was never intended to serve as a reference group. However, there is a trend that those participants who have completed both parts of the blended learning course perform better than those who have not completed both parts.

Furthermore, as a head-to-head comparison between the novel learning approach presented herein and conventional teaching methods was not possible, we performed an “internal quality control” in order to investigate a possible change of knowledge of the participants. To this end, we compared the official examination results of the year in which our study took place to examination results from the previous year. The results from this analysis are provided in the Supplementary material (Internal quality control S6). In summary, there was no statistically significant difference regarding the examination results.

## 4 Discussion

This feasibility study showed the successful design of a blended learning approach in thoracic radiology and its implementation into a pre-existing radiology curricular course. Furthermore, it revealed medical students’ positive attitudes toward this approach and showed an increased knowledge in thoracic radiology. As the best way of transferring knowledge in medical education is still unclear, such approaches might be used to enrich the teaching armamentarium and to further enhance interest and knowledge in thoracic diseases among medical students.

One explanation for these positive results might be the increased flexibility of self-determined learning, and our results are in line with this notion. Students appreciated the broad online course content, which they could use anywhere at any time and at their own pace without having to attend a class. Another point is that the flexibility of self-determined learning could be particularly important in meeting the needs of an increasingly diverse student community (41). The maximal learning and teaching flexibility provided by such courses could accommodate, for example, students with disabilities and thus further enhance inclusivity (41).

On the other hand, from students’ perspective, it requires higher stringency, commitment and intrinsic motivation as they - as first - have to obtain knowledge at their own (21). This includes, e.g., watching prerecorded lectures and/or completing online education modules (17). As this is an additional learning burden compared to traditional classroom teaching, approval among students may soon disappear. And in fact, previous studies suggest that both medical students and lecturers had difficulty motivating themselves (or the students, respectively) to follow online courses (2, 9). In order to be successful, thus, students’ motivation to engage with it is crucial. Possible solutions to this include, e.g., learning content that is designed “activating” as possible by, e.g., videos or short quizzes. Furthermore, a structured framework including learning goals and timetables for each learning module are necessary to guide students through the information jungle (14). These steps are necessary to improve students learning behavior in a successful and self-determined manner and to avoid leaving students behind. Interestingly, our results indicate that students were additionally motivated to become involved with thoracic radiology after using our modules. They even plan to use the modules in the future in order to refresh their knowledge of the subject. Such a refresher might be of particular interest before clerkships in, for example, pulmonology or thoracic surgery.

From lecturers’ perspective, the prior onsite course was changed from instructor-centered teaching to student-centered learning. This step can be somewhat critical and requires high commitment as lecturers have to face new requirements and “evolve” from being the (only) source of information to being a “guide on the side” helping students in the “information jungle” (14). However, this step is most crucial to further enhance students’ learning experience, to show them that they are benefitting from their own efforts and – thus - to avoid motivational problems on both sides (14, 42).

Taking the above-mentioned steps together, at best, this leads to a maximum effective in-class time in which students and teachers can really focus on core topics that are, e.g., difficult to understand and need more time for explanation. From students’ perspective, this might result in an enhanced learning effect.

Despite the possible benefits of a blended learning approach, there is no doubt that the implementation of new teaching concepts require additional effort (43, 44). This is especially true for the transition from traditional, onsite teaching to online or hybrid teaching. First, IT infrastructure has to be installed or adjusted to online teaching. Mostly, its installation results in both a time-intensive and expensive process depending on the local conditions of the institution (43). Regarding the course on thoracic radiology presented herein, radiology departments might have an advantage compared to other specialties as solid IT-solutions or IT-infrastructure might be already available making the switch to online teaching easier.

Furthermore, faculty and lecturers are required to adapt the teaching concept to an online format which is again time-consuming. However, once the material has been made suitable for an online module, it has to be updated periodically, but the lecturer does not have to present the same lecture over and over again. Regarding content creation, a clearly defined curriculum including time tables and learning goals helps here as mentioned above (14). Furthermore, triggered by the pandemic, there is a bouquet of online available teaching resources including online teaching platforms as well as a wide variety of national and international guidelines which can be used for content creation (30, 31). For example, the German Radiological Society launched an interactive online learning platform providing a broad variety of online courses comprising prerecorded lectures, case-based training, educational imaging data, etc., for medical students as well as professionals.^3^ Such innovative teaching projects not only allow for broad “knowledge on demand” but may also enhance the harmonization of radiological teaching content in the future. This is especially true when it comes to inter-faculty implementation of a certain curriculum involving more than one medical school as such tools might provide a certain level of standardization.

Results from previous studies suggest that there is a trend is toward digital learning and teaching in medical education (2, 9, 23, 26, 45). However, there is lots of room for improvement and constant further development is crucial.

### 4.1 Limitations

Due to the specific course design within the framework of a regular curricular radiology course and the curricular regulations of the faculty a head-to-head comparison between our blended learning approach and conventional classroom teaching was not possible. Consequently, statements regarding the superiority of our learning approach compared to conventional approaches are limited. However, an “internal quality control” regarding a possible change of knowledge on the objective test results was performed. In summary, there was no statistically significant difference regarding the examinations results [see also point 3.3 of our results section as well as our Supplementary material (Internal quality control S6)]. Despite these results, however, it must be said that the comparability is limited, and it is difficult to draw conclusions from this: (1) Examinations used for this quality control do not only contain thoracic radiology questions but represent the entire spectrum of the curricular radiology course and (2) the groups including their learning environment are not equal.

Furthermore, this study is questionnaire-based and subject to typical pitfalls. We had to consider selection bias, meaning that interested participants were more likely to complete the questionnaire (46). However, as courses were delivered to all fourth-year medical students, there was no preselection of the participants. Furthermore, the high response rate of our study implies that the pool of participants was representative of the students. Second, there was a potential for social desirability bias, meaning that participants chose the answer they assumed to be favorable (47). In order to attenuate this, we chose an anonymous, untraceable study design and informed the participants that the results were for research purposes only. Due to the specific course design, the onsite course always followed the online part. Thus, we cannot exclude position bias, meaning that participants might have been influenced by the sequence in which the content was presented (48). Furthermore, this blended learning approach was integrated into a pre-existing curricular radiology course which might have affected participants’ knowledge and attitudes toward our blended learning course due to “surrounding” learning activities. Regarding technical issues, the tools used by the participants for their online teaching were not standardized and this use of different tools may have affected the results.

Due to the short follow-up of 2 weeks, this study can only provide a snapshot as we did not examine long-terms effects. Further longitudinal assessments several months or even years after the study would be valuable to examine effects on long-term retention, a possible impact on the development of clinical skills and the practical application of knowledge. As this study included only medical students as participants, such follow-up studies could, for example, also focus on lecturers’ attitudes toward this teaching concept. Such an approach might reveal possible differences in students’ and lecturers’ attitudes toward possible teaching concepts in the future. As this is a single-center study with a specific cohort of students, its findings may not be easily generalizable to other medical schools or student populations limiting the external validity of the study. Thus, further studies should include different medical schools from different nations to investigate the potential of this teaching concept in order to help harmonizing medical curricula around the world.

Finally, this study mainly focused on the assessment of knowledge gain as primary learning objective. However, besides the acquisition of knowledge and practical skills there is an ongoing discourse including fundamental aspects of medical education, of what constitutes effective learning and of the professional attributes required of doctors (49). As medical educators should prepare learners in the best way for their future professional roles it is crucial to expand our perspectives about learning and to consider several relevant learning theories as the foundation of teaching and learning approaches in medical education (4, 49).

## 5 Conclusion

This feasibility study showed the successful design and implementation of a blended learning approach in thoracic radiology into a pre-existing radiology teaching curriculum. First, our study revealed medical students’ great interest in and acceptance of this learning approach. Second, the learning approach presented herein led to an increased knowledge in an objective test in thoracic radiology. Thus, approaches as the one presented herein might further stimulate the evolution of teaching and learning in medical education. However, further studies including, e.g., head-to-head comparisons with conventional teaching approaches as well as studies focusing on lecturers’ attitudes toward new learning approaches are necessary to generate more evidence regarding these novel teaching and learning approaches.

## Data availability statement

Data cannot be shared publicly because of institutional and national data policy restrictions imposed by the Ethics committee since the data contain potentially identifying study participants’ information. Data are available upon request (contact via weimer@uni-mainz.de) for researchers who meet the criteria for access to confidential data (please provide the manuscript title with your enquiry).

## Ethics statement

The studies involving humans were approved by Ethik-Kommission bei der Landesärztekammer Rheinland-Pfalz. The studies were conducted in accordance with the local legislation and institutional requirements. The participants provided their written informed consent to participate in this study.

## Author contributions

FS: Conceptualization, Data curation, Formal analysis, Funding acquisition, Investigation, Methodology, Project administration, Resources, Software, Supervision, Validation, Visualization, Writing – original draft, Writing – review & editing. YY: Conceptualization, Data curation, Formal analysis, Funding acquisition, Investigation, Methodology, Project administration, Resources, Software, Supervision, Validation, Visualization, Writing – original draft, Writing – review & editing. LM: Conceptualization, Data curation, Formal analysis, Funding acquisition, Methodology, Resources, Software, Validation, Visualization, Writing – review & editing. PG: Conceptualization, Data curation, Investigation, Validation, Writing – review & editing. DP: Data curation, Formal analysis, Investigation, Methodology, Software, Supervision, Visualization, Writing – review & editing. PD: Conceptualization, Data curation, Investigation, Supervision, Validation, Writing – review & editing. AW: Data curation, Investigation, Validation, Writing – review & editing. ML: Data curation, Investigation, Validation, Writing – review & editing. RK: Conceptualization, Data curation, Formal analysis, Funding acquisition, Investigation, Methodology, Project administration, Resources, Software, Supervision, Validation, Visualization, Writing – review & editing. JW: Conceptualization, Data curation, Formal analysis, Funding acquisition, Investigation, Methodology, Project administration, Resources, Software, Supervision, Validation, Visualization, Writing – original draft, Writing – review & editing.

## Footnotes

^1^www.surveymonkey.com

^2^https://www.R-project.org

^3^http://conrad.drg.de

## Supplementary material

CHECKLIST S1 Guideline for reporting observational studies.

PRECOURSE EVALUATION S2 Questionnaire investigating various aspects of teaching and learning in radiology and medical education (precourse).

POSTCOURSE EVALUATION S3 Questionnaire investigating various aspects of teaching and learning in radiology and medical education (postcourse).

QUIZ THORACIC RADIOLOGY S4 Test consisting of both multiple-choice and free text questions on thoracic radiology.

SUPPLEMENTARY TABLE S5 Demographic characteristics of the participants.

INTERNAL QUALITY CONTROL S6 Examination results from the year 2021 compared to the examination results from 2022 (the year in which our study took place) (“test”).

SUPPLEMENTARY TABLE S7 Completion rates of the modules (%).

SUPPLEMENTARY TABLE S8 Results of the regression analysis investigating various influencing factors regarding their impact on the objective knowledge test (“posttest”).

SUPPLEMENTARY TABLE S9 Results of the regression analysis investigating various influencing factors regarding their impact on the objective knowledge test (“pretest”).

SUPPLEMENTARY TABLE S10 (Sub-)results of the knowledge tests, broken down by each module separately.

SUPPLEMENTARY FIGURE S1 Overview of the structure of the online learning platform for thoracic radiology.

SUPPLEMENTARY FIGURE S2 Examples of several features which were included to enrich the teaching content of the online learning platform.
