# Supplementary material for: A blended learning approach for teaching thoracic radiology to medical students: a proof-of-concept study
Source: Front Med (Lausanne). 2023 Nov 23;10:1272893. doi: 10.3389/fmed.2023.1272893 (PMC10701891; doi:10.3389/fmed.2023.1272893)
Supplement: PRECOURSE EVALUATION S2 — Questionnaire investigating various aspects of teaching and learning in radiology and medical education (precourse). [file Data_Sheet_2.pdf]

Precourse evaluation S2.Questionnaire investigating various aspects of teaching and learning in radiology and medical education (precourse).

1. Precourse evaluation

1. **Gender**

- ◯ Female
- ◯ Male
- ◯ Other

2. **Age**

[ ]

3. **Personal code**

[ ]

4. **Main subject**

- ☐ Human medicine
- ☐ Dentistry
- ☐ Other

5. **Current study year**

[ ]

6. **Prior professional experience**

- ☐ Yes - medical
- ☐ Yes - nonmedical
- ☐ No

7. **I regularly use online learning resources:**

strongly disagree	disagree	somewhat disagree	neutral	somewhat agree	agree	strongly agree
○	○	○	○	○	○	○

8. **My personal use of online learning resources (in hours per week):**

I do not use any online learning resources — 10 hours per week — 20 hours per week

9. **In general, I am interested in diagnostic imaging:**

strongly disagree	disagree	somewhat disagree	neutral	somewhat agree	agree	strongly agree
○	○	○	○	○	○	○

10. **I have prior experience in diagnostic imaging (e.g., interpretation):**

Yes	☐
No	☐

11. **If "yes", my previous experience comes from:**

- ☐ Textbooks
- ☐ Scientific journals
- ☐ Interactive live online lectures or seminars ("webinars")
- ☐ Radiology class/course
- ☐ Other

12. **I have already used learning resources for learning chest radiology in the past:**

- ☐ Yes - online learning resources
- ☐ Yes - other learning resources
- ☐ No

13. **I use the following resources the most for learning chest radiology:**

- ☐ Online learning platform
- ☐ Ebooks
- ☐ Apps
- ☐ Textbooks
- ☐ Other
- ☐ None/not applicable

14. **The following resources motivate me the most to learn chest radiology**

Online learning platform	☐
Ebooks	☐
Apps	☐
Textbook	☐
Other	☐

15. **The perfect resource for learning chest radiology is the following:**

online learning platform — textbook

16. **The design of the learning resource is important and motivates me to use it:**

strongly disagree	disagree	somewhat disagree	neutral	somewhat agree	agree	strongly agree
○	○	○	○	○	○	○

17. **Before participating in the course, I have sufficient skills and knowledge regarding:**

	strongly disagree	disagree	somewhat disagree	neutral	somewhat agree	agree	strongly agree
Chest X-ray (technical basics as well as normal and pathological findings)	◯	◯	◯	◯	◯	◯	◯
Chest CT (technical basics as well as normal and pathological findings)	◯	◯	◯	◯	◯	◯	◯
Ultrasonography of the lung (technical basics as well as normal and pathological findings)	◯	◯	◯	◯	◯	◯	◯
Spatial orientation in chest radiology	◯	◯	◯	◯	◯	◯	◯
Anatomical knowledge in chest radiology	◯	◯	◯	◯	◯	◯	◯
Interpretation of chest X-ray	◯	◯	◯	◯	◯	◯	◯
Interpretation of chest CT	◯	◯	◯	◯	◯	◯	◯
Interpretation of ultrasonography of the lung	◯	◯	◯	◯	◯	◯	◯

18. **Online learning in medical education:**

	strongly disagree	disagree	somewhat disagree	neutral	somewhat agree	agree	strongly agree
In general, I am satisfied with the online learning resources provided before the pandemic	◯	◯	◯	◯	◯	◯	◯
In general, I am satisfied with the online learning resources currently provided	◯	◯	◯	◯	◯	◯	◯
In the future, I would like more online learning offerings	◯	◯	◯	◯	◯	◯	◯

19. **This course on chest radiology will hopefully help me to...**

	strongly disagree	disagree	somewhat disagree	neutral	somewhat agree	agree	strongly agree
get a better theoretical and practical knowledge of chest radiology	◯	◯	◯	◯	◯	◯	◯
get a better anatomical knowledge of chest radiology	◯	◯	◯	◯	◯	◯	◯
improve my skills in the interpretation of X-ray, CT and ultrasonography	◯	◯	◯	◯	◯	◯	◯
improve my skills in differentiating between normal and pathological findings	◯	◯	◯	◯	◯	◯	◯
improve my knowledge of how to make a correct diagnosis	◯	◯	◯	◯	◯	◯	◯
