# Supplementary material for: A blended learning approach for teaching thoracic radiology to medical students: a proof-of-concept study
Source: Front Med (Lausanne). 2023 Nov 23;10:1272893. doi: 10.3389/fmed.2023.1272893 (PMC10701891; doi:10.3389/fmed.2023.1272893)
Supplement: POSTCOURSE EVALUATION S3 — Questionnaire investigating various aspects of teaching and learning in radiology and medical education (postcourse). [file Data_Sheet_3.pdf]

Postcourse evaluation S3. Questionnaire investigating various aspects of teaching and learning in radiology and medical education (postcourse).

1. Postcourse evaluation

1. **Gender**

- ◯ Female
- ◯ Male
- ◯ Other

2. **Age**

☐

3. **Personal code**

☐

4. **Main subject**

- ☐ Human medicine
- ☐ Dentistry
- ☐ Other

5. **Current study year**

☐

6. **Prior professional experience**

- ☐ Yes - medical
- ☐ Yes - nonmedical
- ☐ No

7. **I regularly use online learning resources:**

strongly disagree	disagree	somewhat disagree	neutral	somewhat agree	agree	strongly agree
○	○	○	○	○	○	○

8. **My personal use of online learning resources (in hours per week):**

I do not use any online learning resources — 10 hours per week — 20 hours per week

9. **In general, I am interested in diagnostic imaging:**

strongly disagree	disagree	somewhat disagree	neutral	somewhat agree	agree	strongly agree
○	○	○	○	○	○	○

10. **I have prior experience in diagnostic imaging (e.g., interpretation):**

Yes	☐
No	☐

11. **If "yes", my previous experience comes from:**

- ☐ Textbooks
- ☐ Scientific journals
- ☐ Interactive live online lectures or seminars ("webinars")
- ☐ Radiology class/course
- ☐ Other

12. **I have already used learning resources for learning chest radiology in the past:**

- ☐ Yes - online learning resources
- ☐ Yes - other learning resources
- ☐ No

13. **After completion of the course, I have sufficient skills and knowledge regarding:**

	strongly disagree	agree	somewhat disagree	neutral	somewhat agree	agree	strongly agree
Chest X-ray (technical basics as well as normal and pathological findings)	◯	◯	◯	◯	◯	◯	◯
Chest CT (technical basics as well as normal and pathological findings)	◯	◯	◯	◯	◯	◯	◯
Ultrasonography of the lung (technical basics as well as normal and pathological findings)	◯	◯	◯	◯	◯	◯	◯
Spatial orientation in chest radiology	◯	◯	◯	◯	◯	◯	◯
Anatomical knowledge in chest radiology	◯	◯	◯	◯	◯	◯	◯
Interpretation of chest X-ray	◯	◯	◯	◯	◯	◯	◯
Interpretation of chest CT	◯	◯	◯	◯	◯	◯	◯
Interpretation of ultrasonography of the lung	◯	◯	◯	◯	◯	◯	◯

Postcourse evaluation S3. Questionnaire investigating various aspects of teaching and learning in radiology and medical education (postcourse).

2. Hier Titel einfügen

14. **I particularly used the online learning platform...**

- ☐ during the semester
- ☐ during a clerkship
- ☐ to prepare for examinations
- ☐ for follow-up/post-processing
- ☐ other

15. **Design/layout of the online learning platform:**

	strongly disagree	disagree	somewhat disagree	neutral	somewhat agree	agree	strongly agree
The platform is visually appealing	◯	◯	◯	◯	◯	◯	◯
The website interface is well structured	◯	◯	◯	◯	◯	◯	◯
The menu items are clear and comprehensible	◯	◯	◯	◯	◯	◯	◯
The character size is sufficient	◯	◯	◯	◯	◯	◯	◯
The number of images provided is sufficient	◯	◯	◯	◯	◯	◯	◯
The size and quality of images provided is sufficient	◯	◯	◯	◯	◯	◯	◯
The size of the videos provided is sufficient	◯	◯	◯	◯	◯	◯	◯
In general, the platform is easy to use and intuitive	◯	◯	◯	◯	◯	◯	◯
All in all, the platform is user-friendly	◯	◯	◯	◯	◯	◯	◯

16. **Technical aspects:**

	strongly disagree	disagree	somewhat disagree	neutral	somewhat agree	agree	strongly agree
Accessing the online learning platform worked flawlessly	◯	◯	◯	◯	◯	◯	◯
The technical functions of the platform worked flawlessly	◯	◯	◯	◯	◯	◯	◯
Accessing the modules worked flawlessly	◯	◯	◯	◯	◯	◯	◯
The images were depicted correctly	◯	◯	◯	◯	◯	◯	◯
The video playback was smooth	◯	◯	◯	◯	◯	◯	◯

17. **Content of the online learning platform:**

	strongly disagree	disagree	somewhat disagree	neutral	somewhat agree	agree	strongly agree
The processing time was sufficient	◯	◯	◯	◯	◯	◯	◯
The amount of learning content was sufficient	◯	◯	◯	◯	◯	◯	◯
The level of difficulty was appropriate	◯	◯	◯	◯	◯	◯	◯
The learning content was sufficiently structured	◯	◯	◯	◯	◯	◯	◯
The learning objectives were comprehensible	◯	◯	◯	◯	◯	◯	◯
The learning content was clinically relevant	◯	◯	◯	◯	◯	◯	◯

18. **This course on chest radiology...**

	strongly disagree	disagree	somewhat disagree	neutral	somewhat agree	agree	strongly agree
helped me to get a better theoretical and practical knowledge of chest radiology	◯	◯	◯	◯	◯	◯	◯
helped me to get a better anatomical knowledge of chest radiology	◯	◯	◯	◯	◯	◯	◯
helped me to improve my skills in the interpretation of X-ray, CT, and ultrasonography	◯	◯	◯	◯	◯	◯	◯
helped me to improve my skills in differentiating between normal and pathological findings	◯	◯	◯	◯	◯	◯	◯
improved my knowledge of how to make a correct diagnosis	◯	◯	◯	◯	◯	◯	◯
motivated me to become involved with chest radiology in the future	◯	◯	◯	◯	◯	◯	◯
was useful for my future career	◯	◯	◯	◯	◯	◯	◯

19. **The online learning platform fulfilled my expectations:**

strongly disagree	disagree	somewhat disagree	neutral	somewhat agree	agree	strongly agree
◯	◯	◯	◯	◯	◯	◯

20. **I will use the online learning platform to refresh my knowledge in the future:**

strongly disagree	disagree	somewhat disagree	neutral	somewhat agree	agree	strongly agree
◯	◯	◯	◯	◯	◯	◯

21. **All in all, I rank the course:**

☆ ☆ ☆ ☆ ☆
