# Supplementary material for: A blended learning approach for teaching thoracic radiology to medical students: a proof-of-concept study
Source: Front Med (Lausanne). 2023 Nov 23;10:1272893. doi: 10.3389/fmed.2023.1272893 (PMC10701891; doi:10.3389/fmed.2023.1272893)
Supplement: QUIZ THORACIC RADIOLOGY S4 — Test consisting of both multiple-choice and free text questions on thoracic radiology. [file Data_Sheet_4.pdf]

Quiz thoracic radiology S4. Test consisting of both multiple-choice and free text questions on thoracic radiology.

1. **Which of the following statements regarding chest X-ray is correct?**

- ◯ A: Typically, soft X-ray radiation is used (energy <50 keV).
- ◯ B: Typically, normal X-ray radiation is used (energy 50-100 keV).
- ◯ C: Typically, hard X-ray radiation is used (energy >100 keV).
- ◯ D: Typically, variable X-ray radiation is used (between 50-150 keV), depending on the patient's weight.

2. **Which of the following statements regarding chest X-ray is wrong?**

- ◯ A: It may be performed with the patient lying down or upright.
- ◯ B: In a posteroanterior (PA) view, the x-ray beam enters through the anterior aspect of the chest and exits out of the posterior.
- ◯ C: In the lateral view, the patient stands with both arms raised and the left side of the chest pressed against the detector.
- ◯ D: In anteroposterior (AP) views, the x-ray beam enters through the anterior aspect and exits through the posterior aspect of the chest.

3. **Which of the following statements regarding chest CT is <u>correct</u>?**

- ◯ A: A 3D reconstruction is feasible.
- ◯ B: In modern CT scanners, the patient is moved axially at a uniform rate during gantry rotation.
- ◯ C: Typically, hard X-ray radiation is used (energy 50-80 keV).
- ◯ D: More than one X-ray beam source at a time is needed.

4. **Which of the following statements is <u>wrong</u>?**

- ◯ A: HU is an abbreviation for Hounsfield unit.
- ◯ B: The attenuation coefficient is a measure of how easily materials can be penetrated by X-rays.
- ◯ C: In clinical routine, a Hounsfield scale ranging from -1000 to +3000 is typically used.
- ◯ D: Typically, air is defined as -100 HU and water as +100 HU.

5. **Please name the correct windowing for both CT scans:**

A: [ ]

B: [ ]

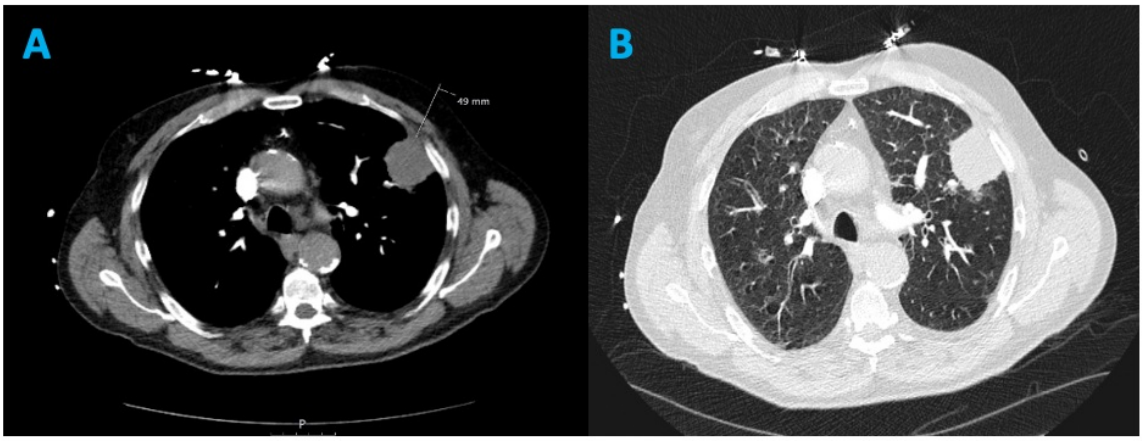

6. **Which of the following statements regarding ultrasonography is <u>wrong</u>?**

◯ A: The lower the frequency, the higher the depth of penetration.

◯ B: Posterior sound transmission typically occurs when investigating bony structures.

◯ C: Edge shadowing arises due to refraction of the beam.

◯ D: Echogenicity is influenced by the density of a tissue.

7. **Please fill in the most fitting anatomical terms (1-4):**

1: [ ]

2: [ ]

3: [ ]

4: [ ]

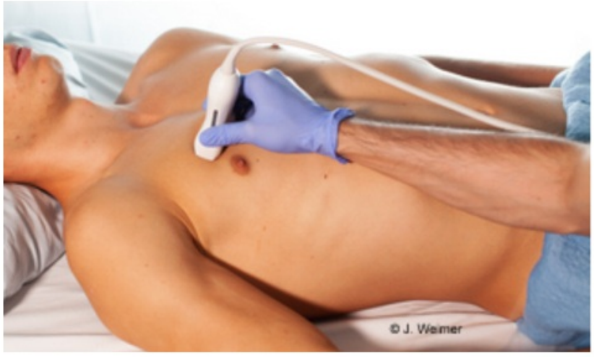

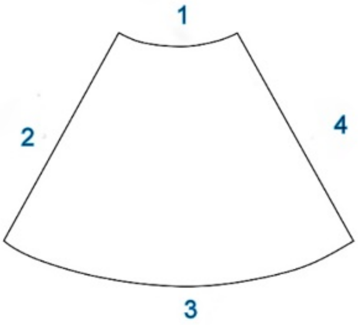

8. **Which imaging modes are depicted in the ultrasonograms depicted below?**

A: [ ]

B: [ ]

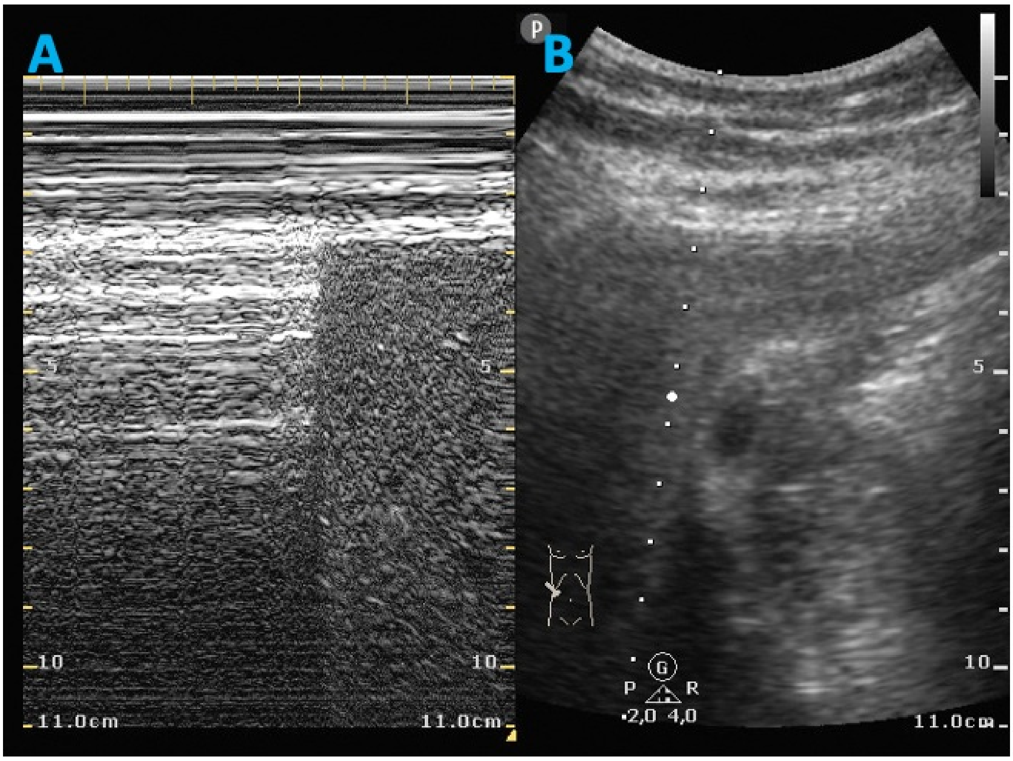

9. **Which of the following statements/markings is <u>correct</u>?**

- ◯ A. Yellow: trachea; red: aortic arch; green: left ventricle; blue: colon.
- ◯ B. Yellow: trachea; red: ascending aorta; green: right ventricle; blue: stomach
- ◯ C. Yellow: oesophagus; red: aortic arch; green: left ventricle; blue: colon
- ◯ D. Yellow: oesophagus; red: ascending aorta; green: right ventricle; blue: stomach

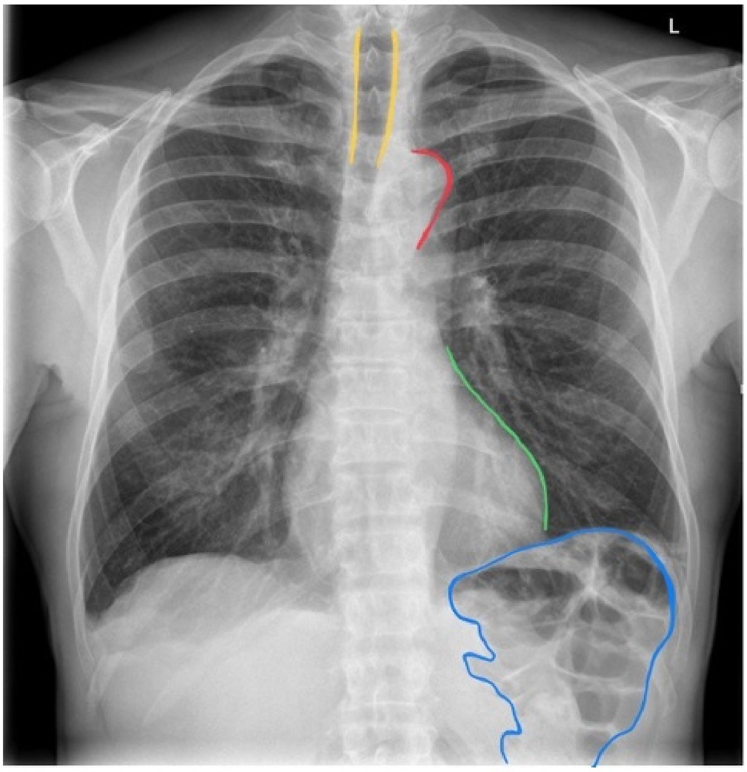

10. **Which of the following statements/markings is <u>correct</u>?**

- ◯ A. Yellow: oesophagus; red: trachea; green: left ventricle; blue: colon
- ◯ B. Yellow: oesophagus; red: trachea; green: right ventricle; blue: stomach
- ◯ C. Yellow: trachea; red: oesophagus; green: right ventricle; blue: colon
- ◯ D. Yellow: trachea; red: oesophagus; green: left ventricle; blue: stomach

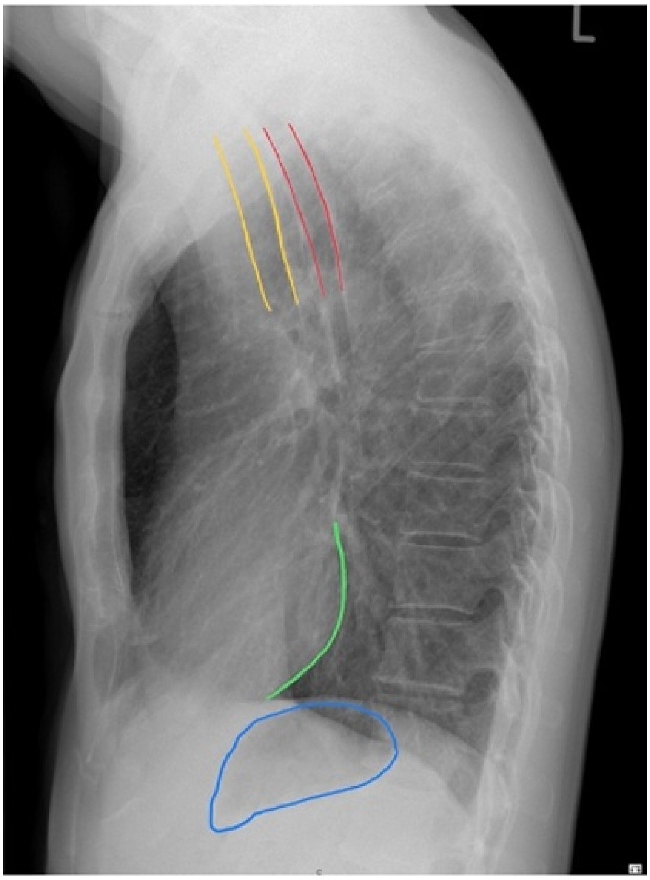

11. **Which of the following statements/markings is <u>correct</u>?**

◯ A. Green: right middle lobe; blue: superior vena cava

◯ B. Green: right middle lobe; blue: inferior vena cava

◯ C. Green: right upper lobe; blue: superior vena cava

◯ D. Green: right upper lobe; blue: inferior vena cava

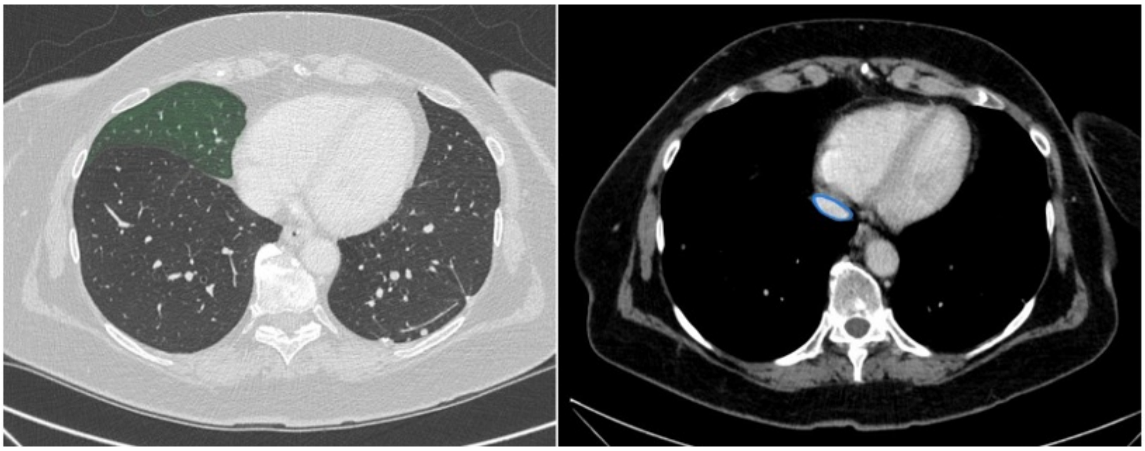

12. **Which of the following statements/markings is <u>correct</u>?**

- ◯ A. Blue: brachiocephalic trunk; red: arteria lusoria
- ◯ B. Blue: superior vena cava; red: vena azygos
- ◯ C. Blue: superior vena cava; red: arteria lusoria
- ◯ D. Blue: brachiocephalic trunk; red: vena azygos

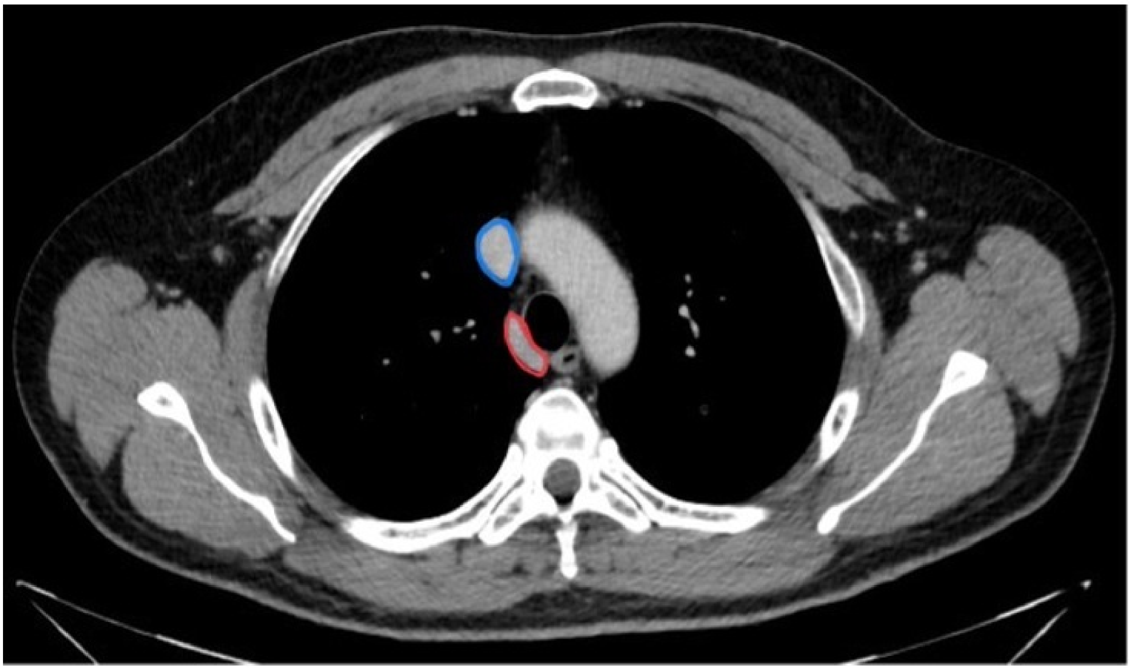

13. **Please name the structures marked below:**

**Red:**	
**Green:**	
**Yellow:**	

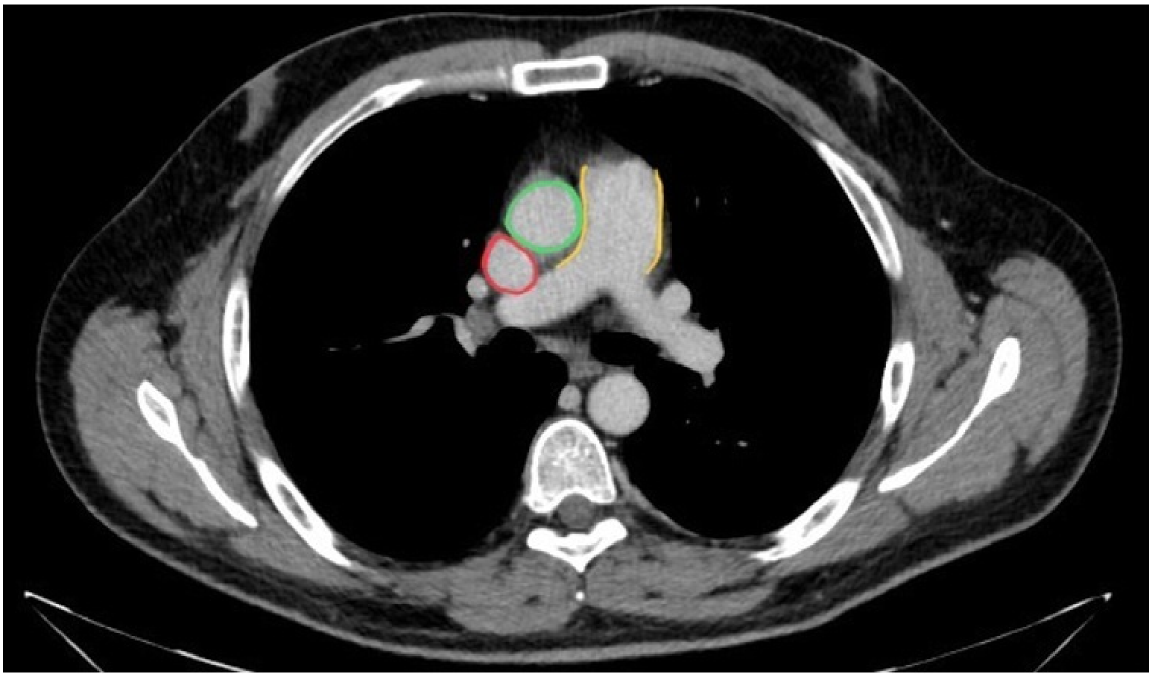

14. **Please name the structures/artifacts in the ultrasonogram below:**

**1:**	
**2:**	
**3:**	
**4:**	

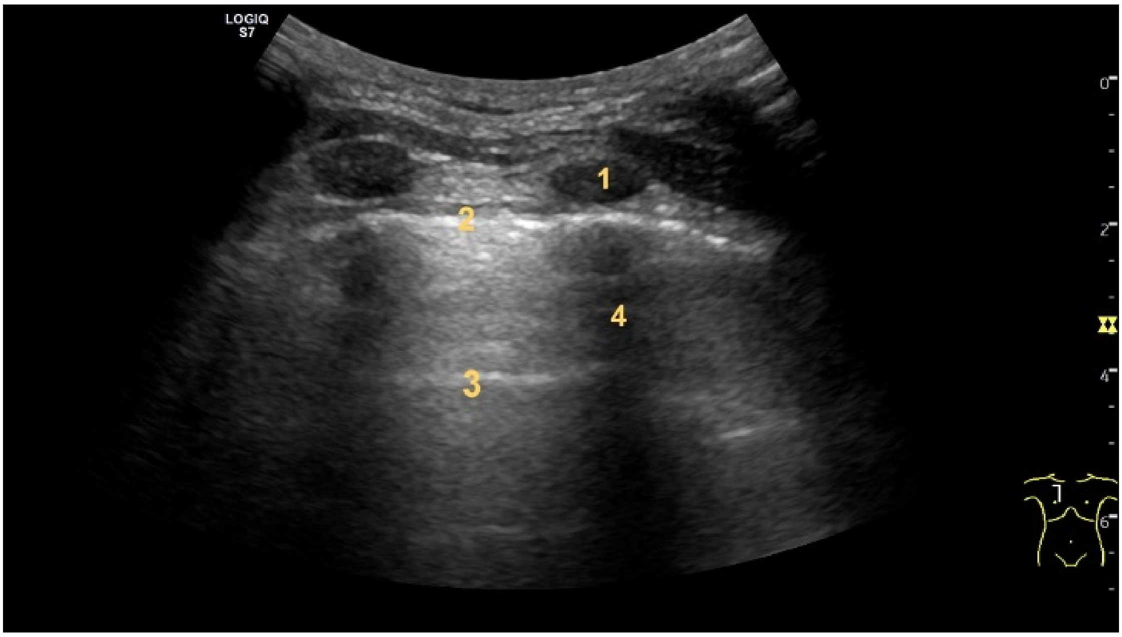

15. **Please name the structures/artifacts in the drawings depicted below:**

1:

2:

3:

4:

5:

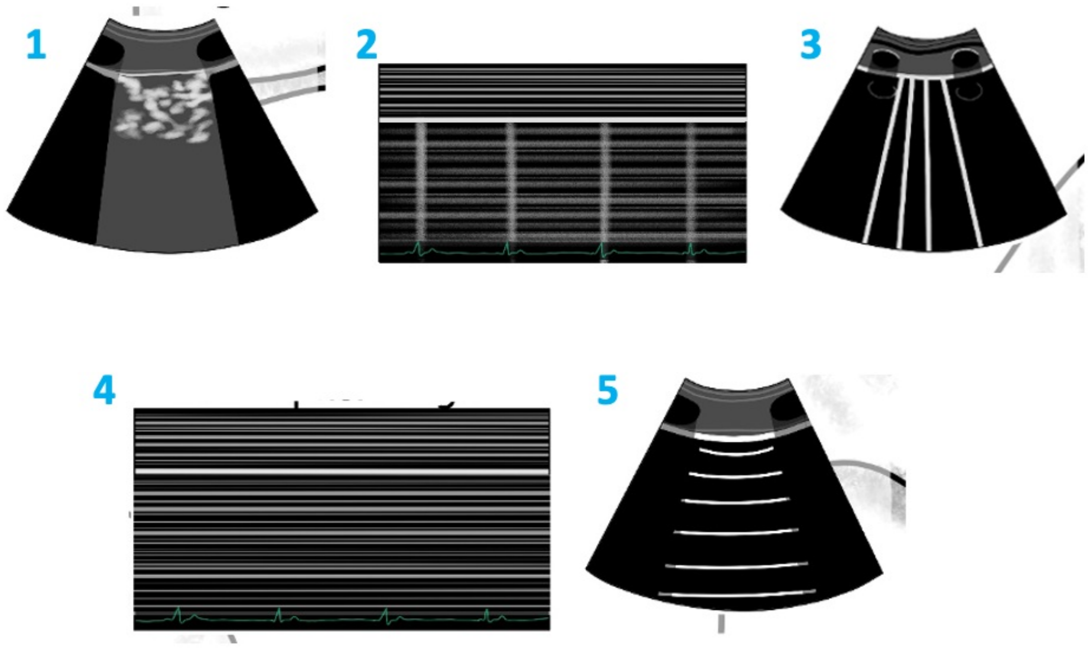

16. **Which of the following statements is <u>correct</u>?**

1=heart 2=diaphragm
3=spleen 4=bowel

- A. 1
- B. 2
- C. 3
- D. 4

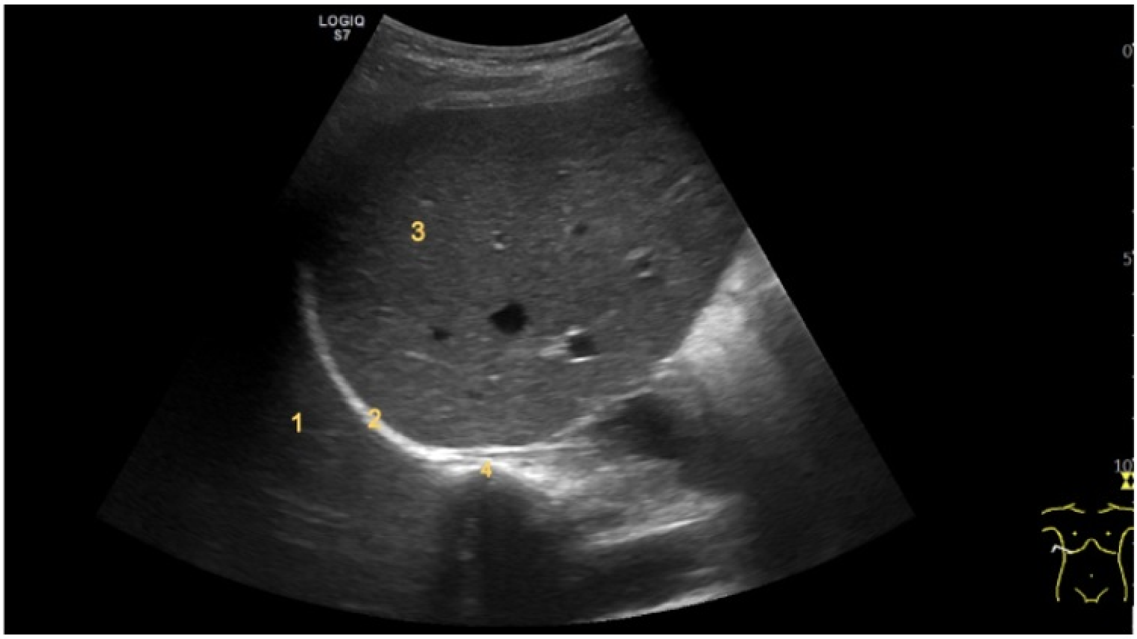

17. **Which of the following statements is <u>correct</u>?**

- ◯ A: Homogeneous lucency over both lungs (infiltration).
- ◯ B: Homogeneous density over both lungs (pleural effusion).
- ◯ C: Homogeneous lucency over both lungs (pleural effusion).
- ◯ D: Homogeneous density over both lungs (infiltration).

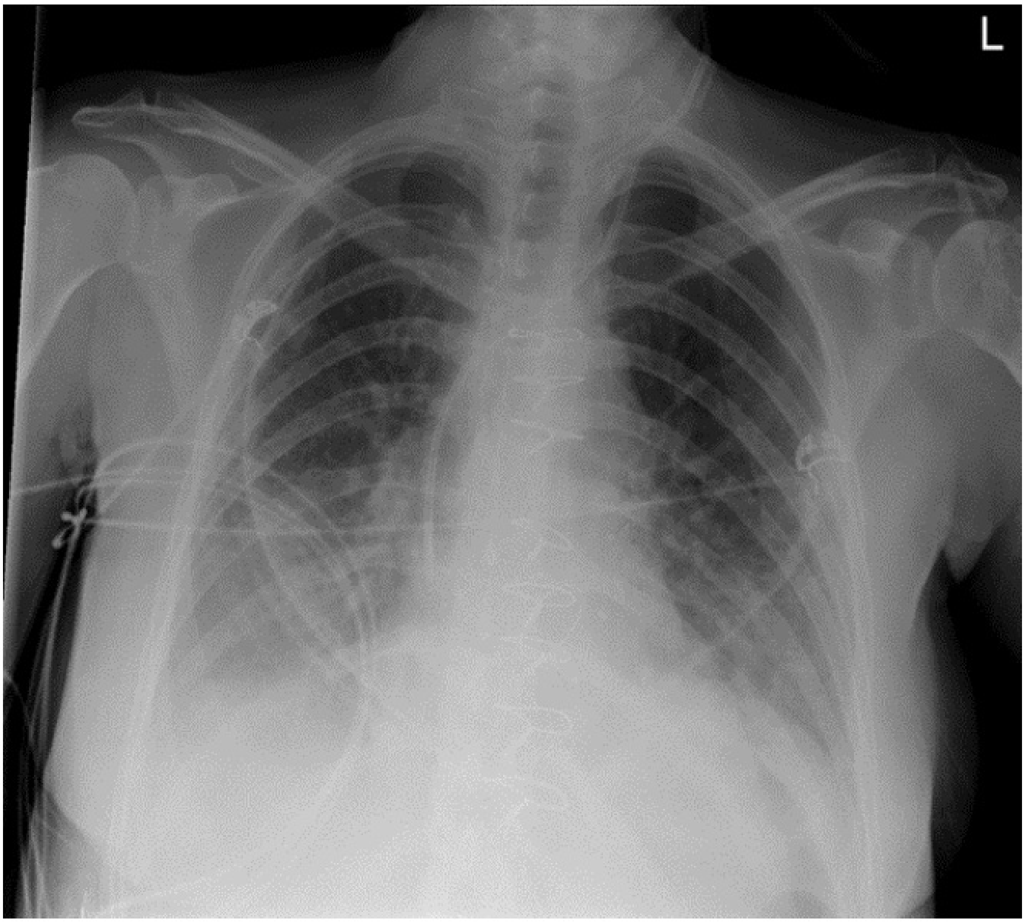

18. **Patient presents with dyspnea, fever, and increased inflammatory parameters.**

Which of the following statements is <u>correct</u>?

- ◯ A: Density in the left lower lobe, partially including a positive air bronchogram, indicating atelectasis.
- ◯ B: Density in the left lower lobe, including pleural effusion and a positive air bronchogram, indicating infiltration.
- ◯ C: Density in the left lower lobe indicating pleural effusion and pneumothorax (i.e., sero-pneumothorax).
- ◯ D: Lucency in the left lower lobe, indicating pneumothorax.

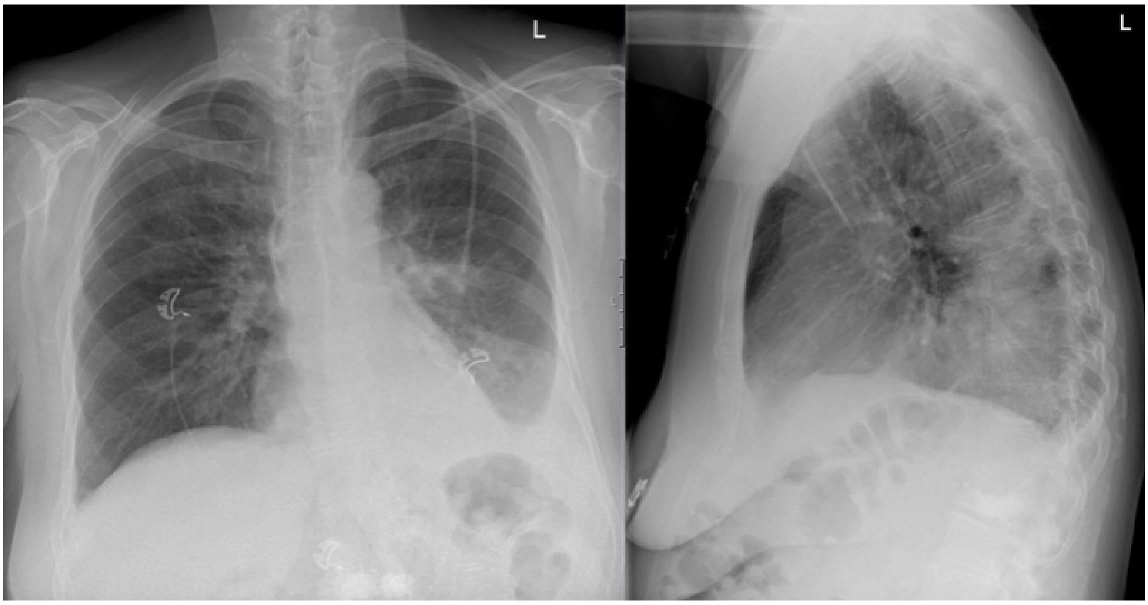

19. **Patient presents with coughing and dyspnea.**

Which of the following statements is <u>correct</u> (multiple answers might be correct)?

- [ ] A: Density in the right upper lobe indicating infiltration.
- [ ] B: Density in the right upper lobe indicating a tumour.
- [ ] C: Density in the right upper lobe indicating a rounded atelectasis.
- [ ] D: Enlargement of the right hilar region indicating hilar lymph node enlargement.

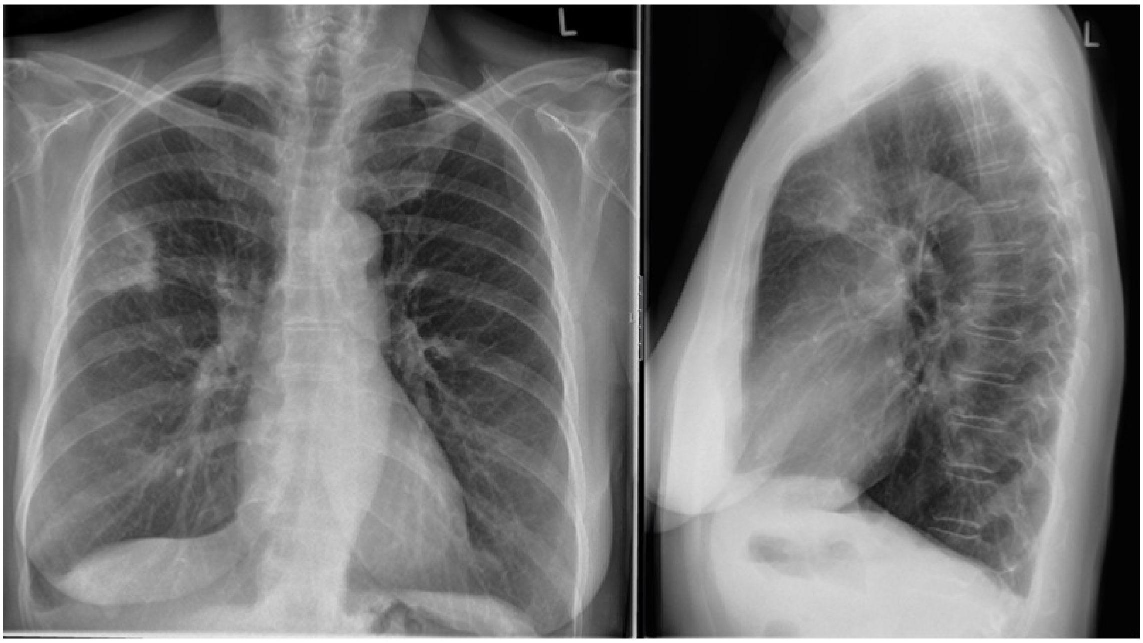

20. **Which of the following statements is <u>correct</u> (multiple answers might be correct)?**

- ☐ A: No pathological findings.
- ☐ B: Emphysema.
- ☐ C: Multiple, perihilar lung metastases (both lungs).
- ☐ D: Perihilar infiltration (both lungs).

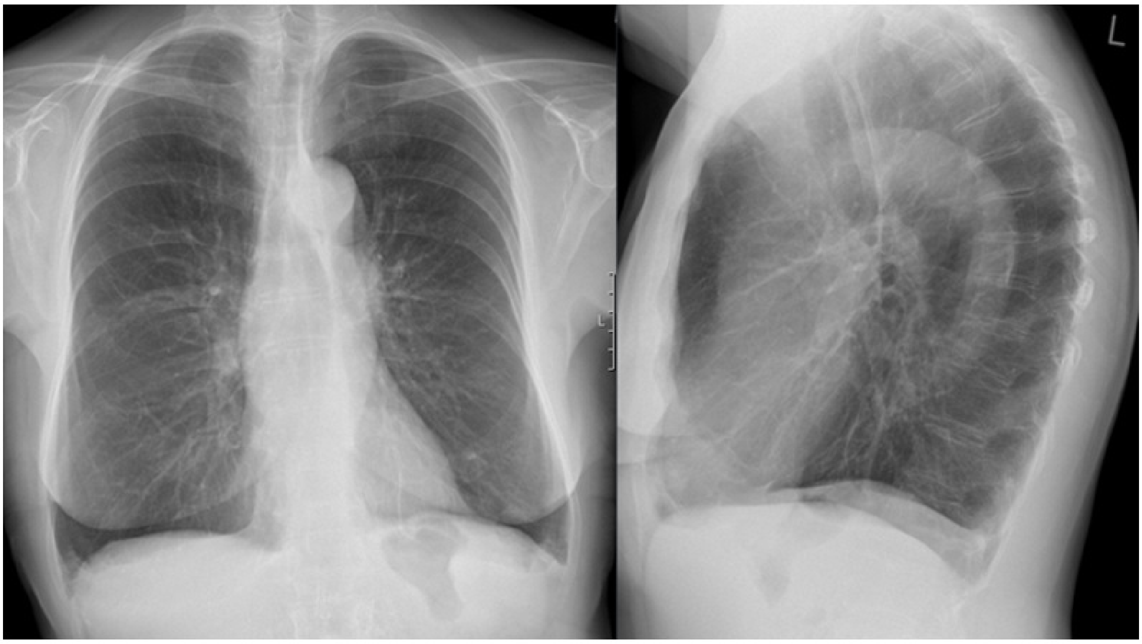

21. **Which of the following statements is correct (multiple answers might be correct)?**

- [ ] A: Bilateral pleural effusion.
- [ ] B: Left-sided pneumothorax.
- [ ] C: Hiatus hernia.
- [ ] D: Chest X-ray post cardiothoracic surgery.

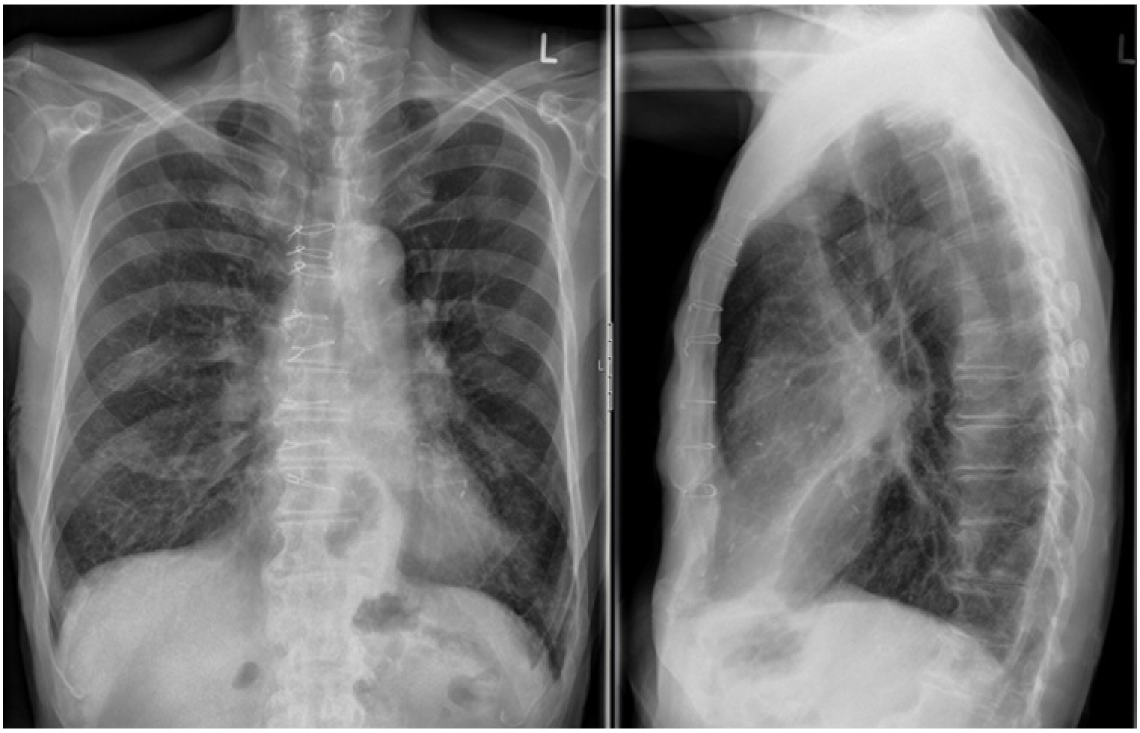

22. **Which of the following statements/diagnoses is correct (multiple answers might be correct)?**

- [ ] A: Pleural effusion.
- [ ] B: Mediastinal mass.
- [ ] C: Hilar mass.
- [ ] D: Compression of inferior vena cava.

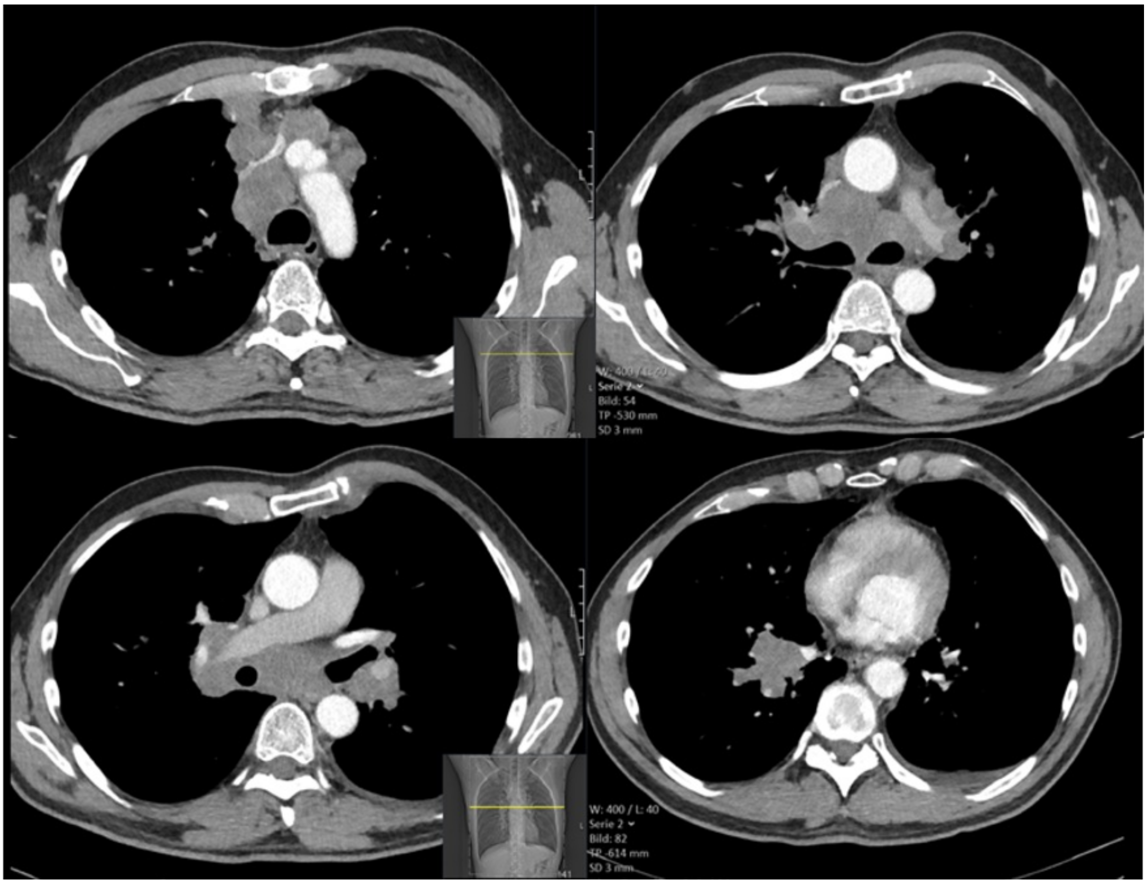

23. **Patient presents with dyspnea and mild fever.**

Which of the following statements is <u>correct</u> (multiple answers might be correct)?

- ☐ A: Image A is reconstructed as a soft tissue window; massive infiltrations in the right upper lobe with pleural effusion.
- ☐ B: Image A is reconstructed as a vessel window; pulmonary embolism in the left pulmonary artery.
- ☐ C: Image B is reconstructed as a lung window; ground-glass opacities (bilateral) and reticulated thickening of interstitial space indicating interstitial/atypical pneumonia.
- ☐ D: Image B is reconstructed as a lung window; ground-glass opacities (bilateral) and reticulated thickening of interstitial space indicating lobar pneumonia.
- ☐ E: Image B is reconstructed as a lung window; ground-glass opacities (bilateral) and reticulated thickening of interstitial space indicating indicating pneumonia due to infarction.

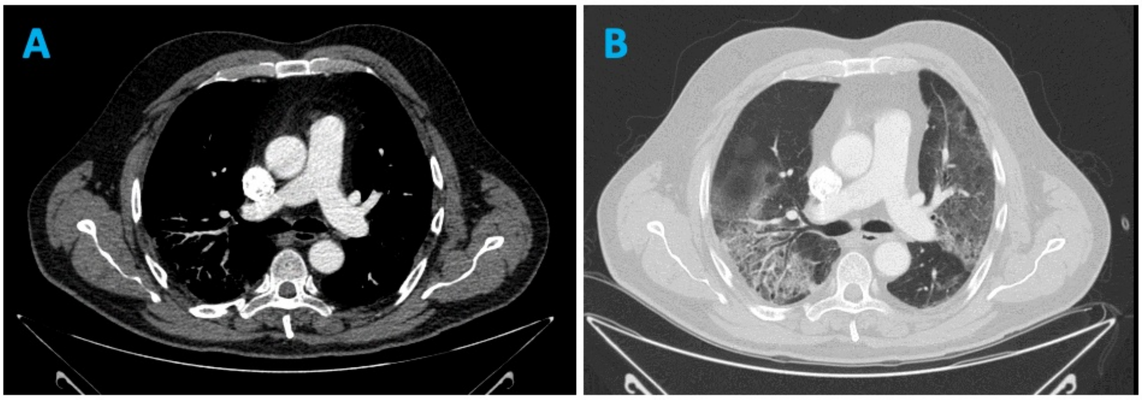

24. **Which of the following CT scans is suggestive for COVID pneumonia?**

- ◯ A
- ◯ B
- ◯ C
- ◯ D

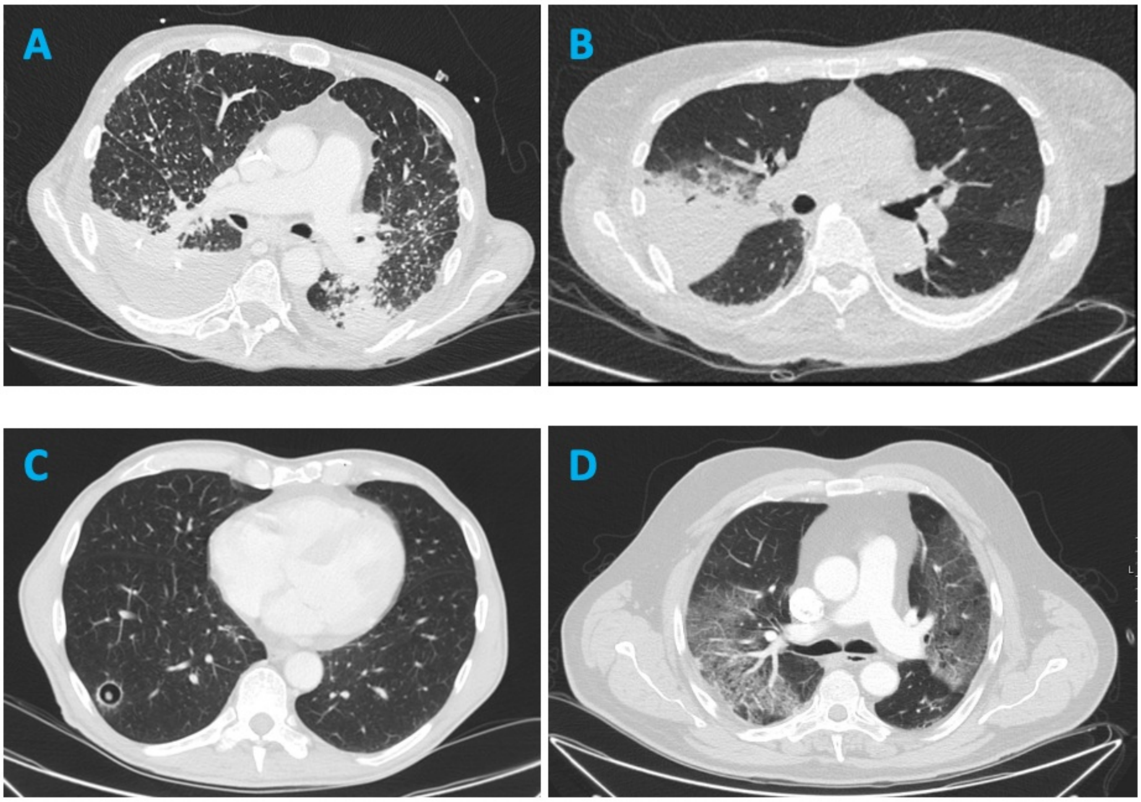

25. **Which of the following statements is <u>correct</u> (multiple answers might be correct)?**

- [ ] A: There is a solid mass in the right middle lobe.
- [ ] B: There is a solid masse in the right lower lobe.
- [ ] C: Mass is spiculated indicating lung cancer.
- [ ] D: Mass is spiculated, indicating tuberculosis.
- [ ] E: HU of -943 is suggestive for emphysema.

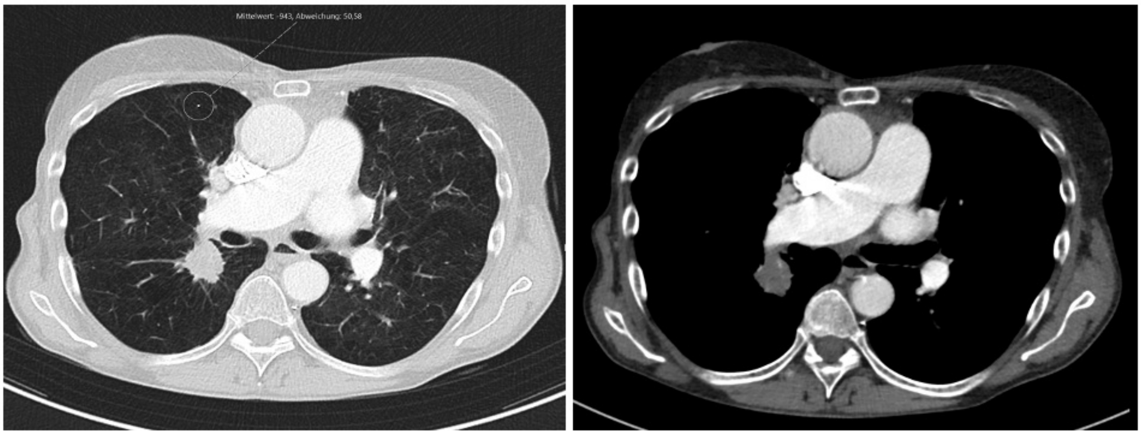

26. **Please name the most fitting pathological finding/diagnosis:**

**A:**	
**B:**	
**C:**	
**D:**	

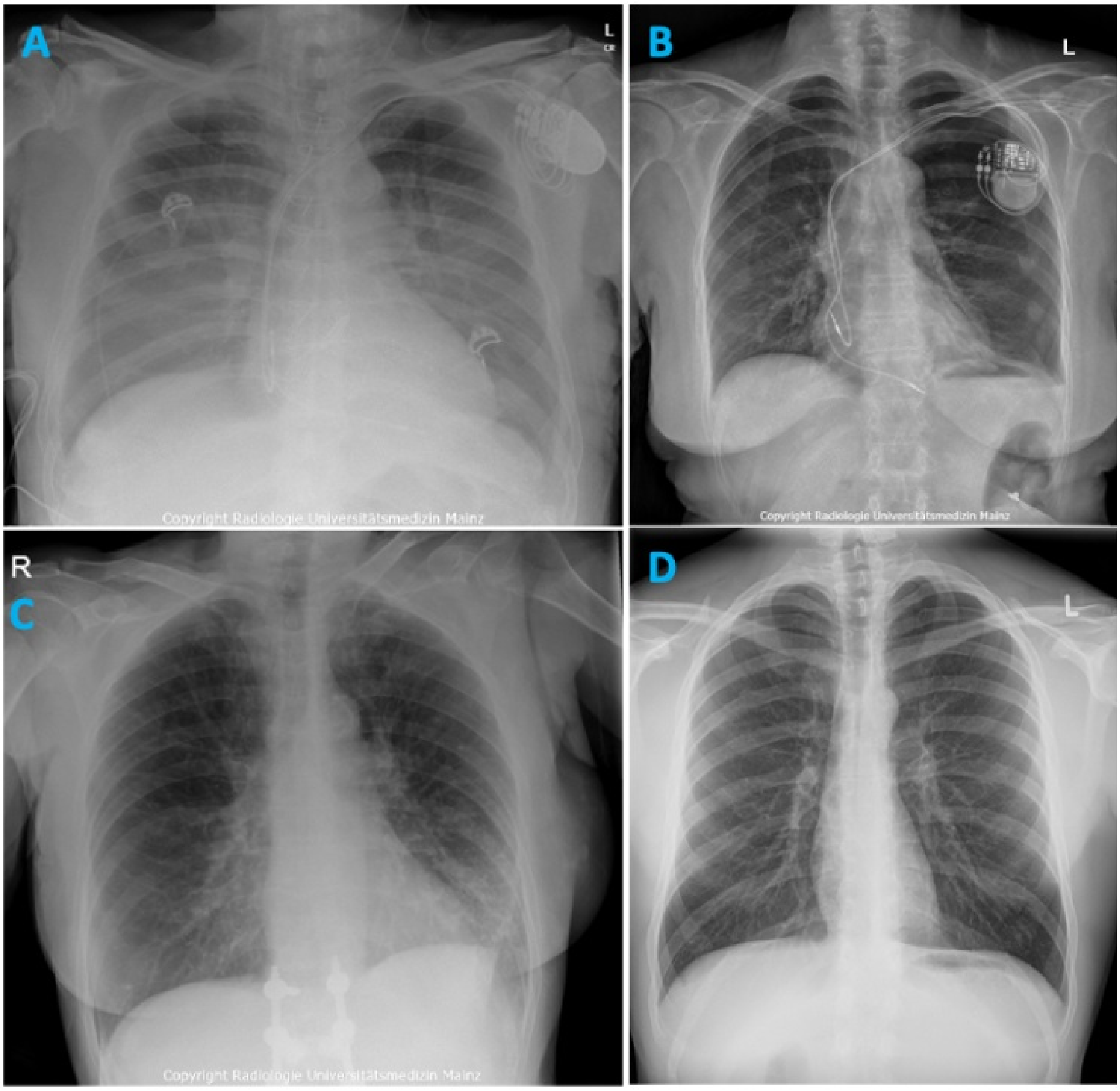

27. **Please name the most fitting pathological finding/diagnosis: (Image B: -973 HU)**

A:

B:

C:

D:

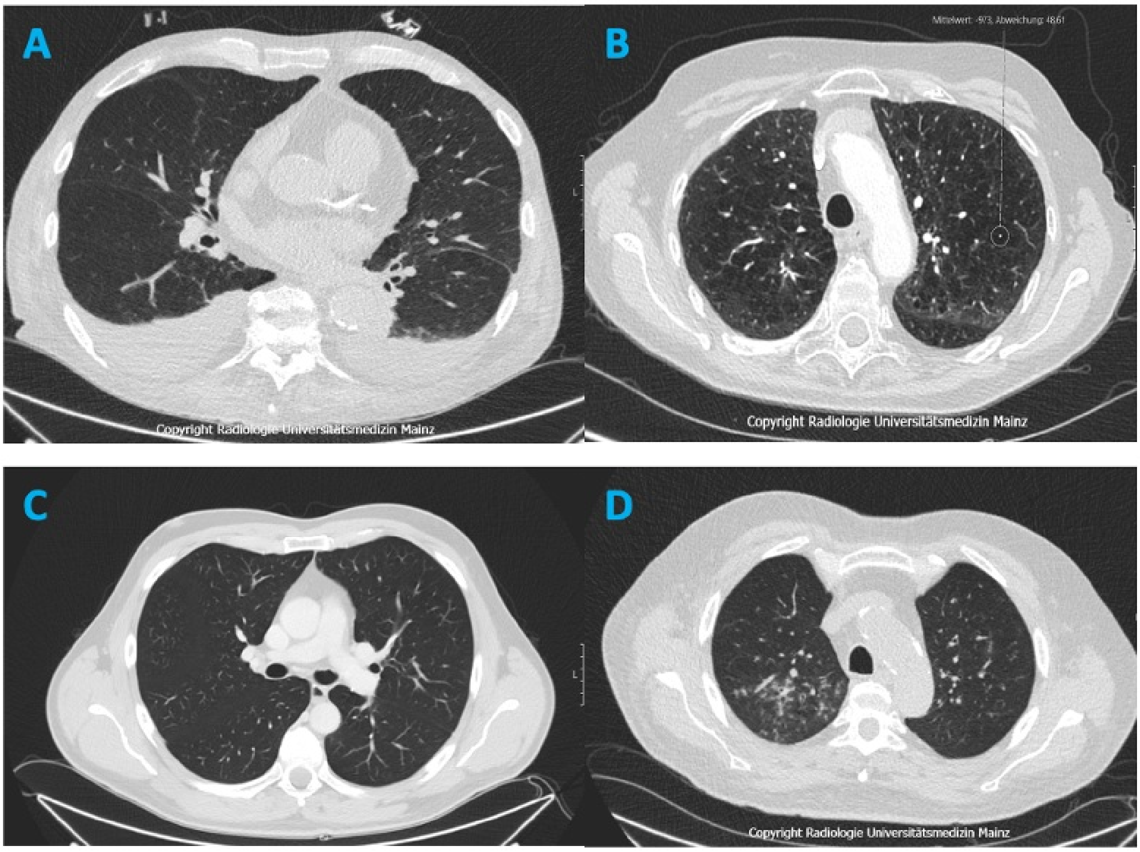

28. **Please assign the following ultrasonography signs to the most fitting pathological finding** (i.a. bat sign, seashore sign, tissue like sign, stratosphere sign, B lines, shred sign, quad sign):

pulmonary edema:

pneumonia:

pleural effusion:

pneumothorax:

29. **Please name the most fitting pathological finding (A-D):**

A:

B:

C:

D:

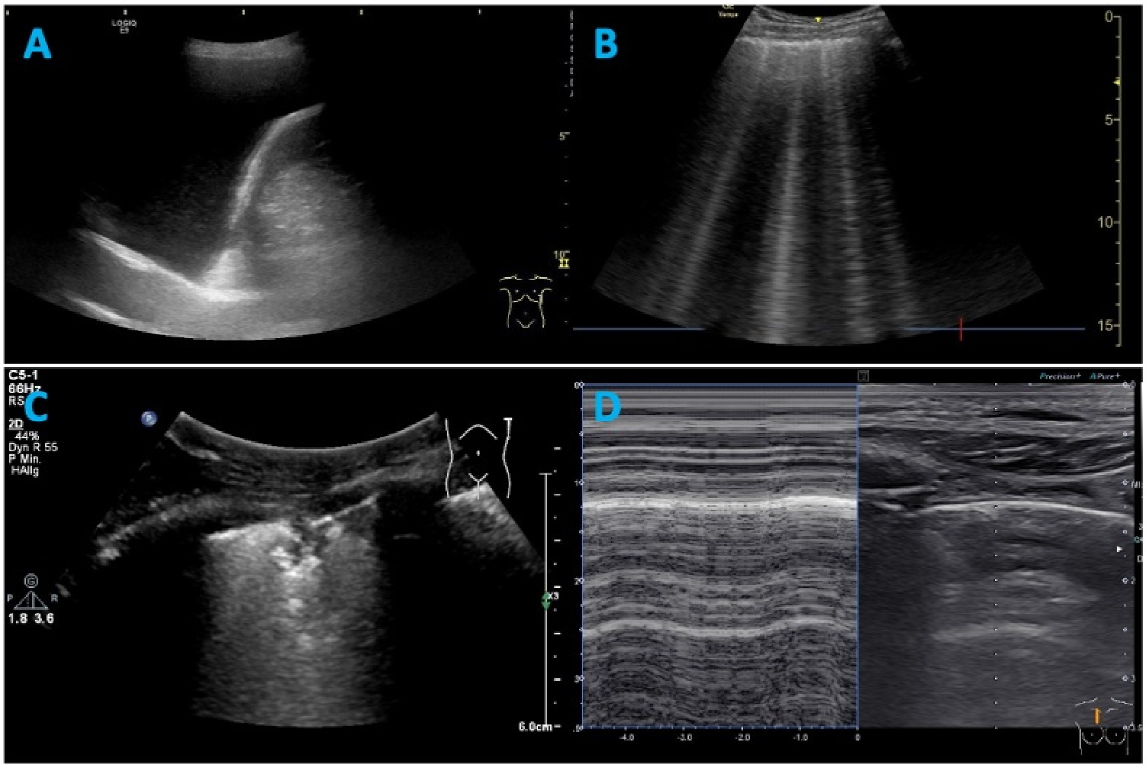

30. **Please assign the most fitting X-ray/CT scan (A-D) to the ultrasonogram depicted below:**

- ◯ A
- ◯ B
- ◯ C
- ◯ D

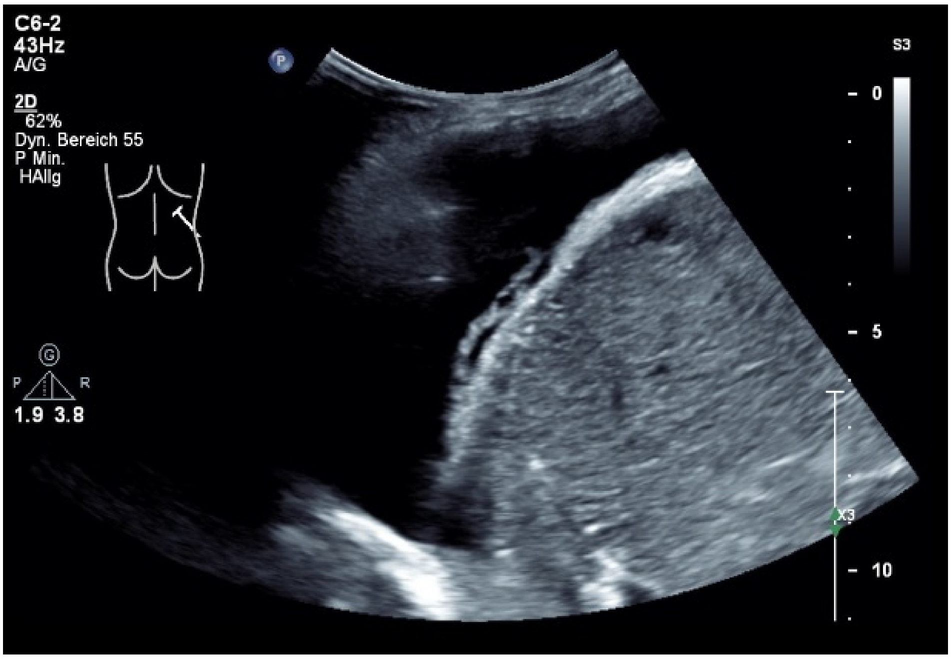

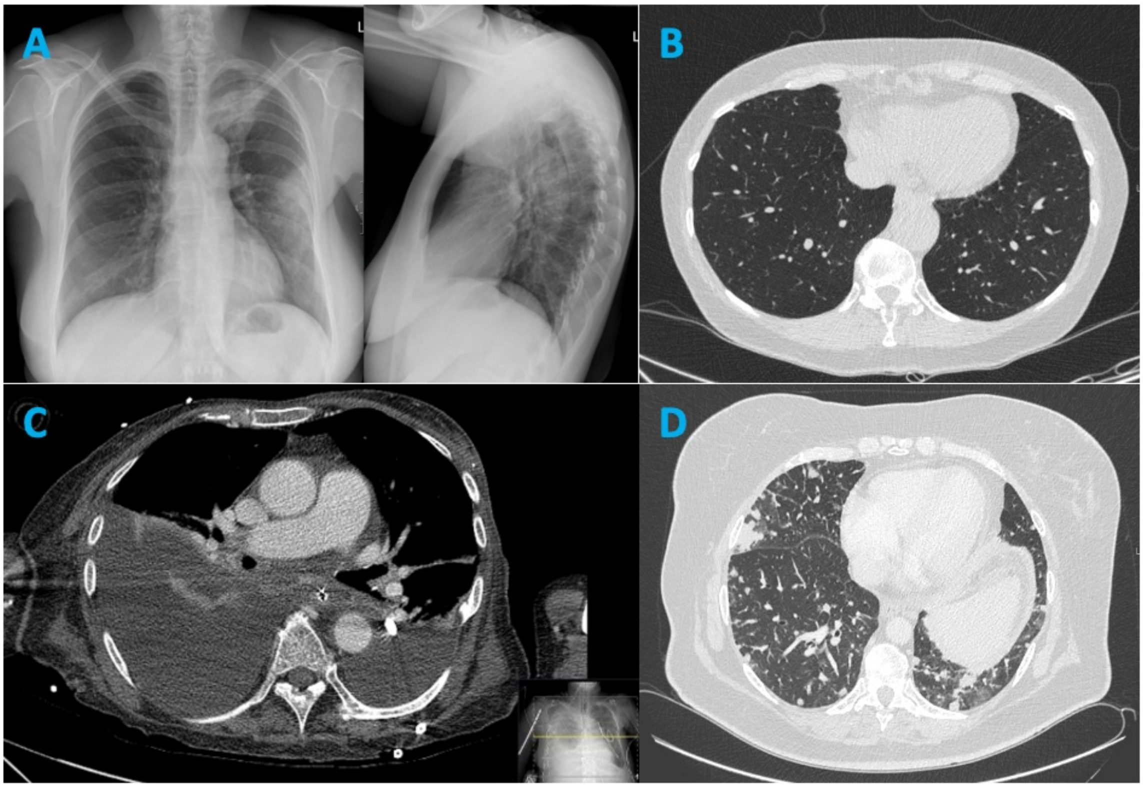

31. **Please assign the most fitting CT scan (A-D) to the ultrasonogram depicted below:**

- ◯ A
- ◯ B
- ◯ C
- ◯ D

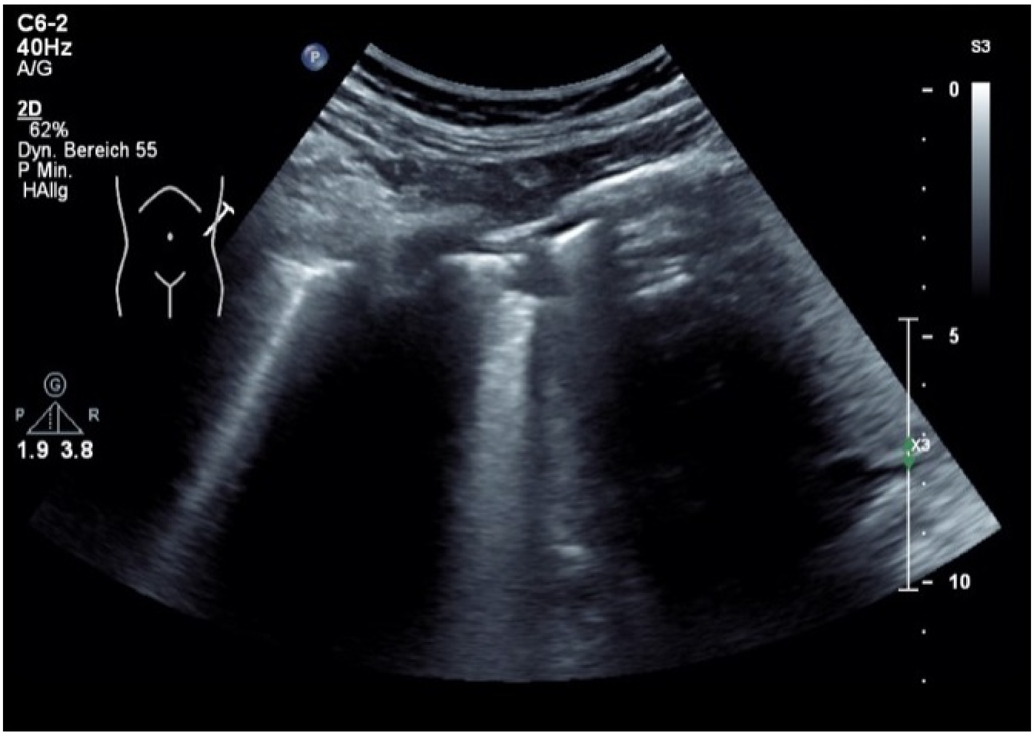
C6-2
40Hz
A/G
2D
62%
Dyn. Bereich 55
P Min.
HAllg
S3
0
5
10
P
R
1.9 3.8

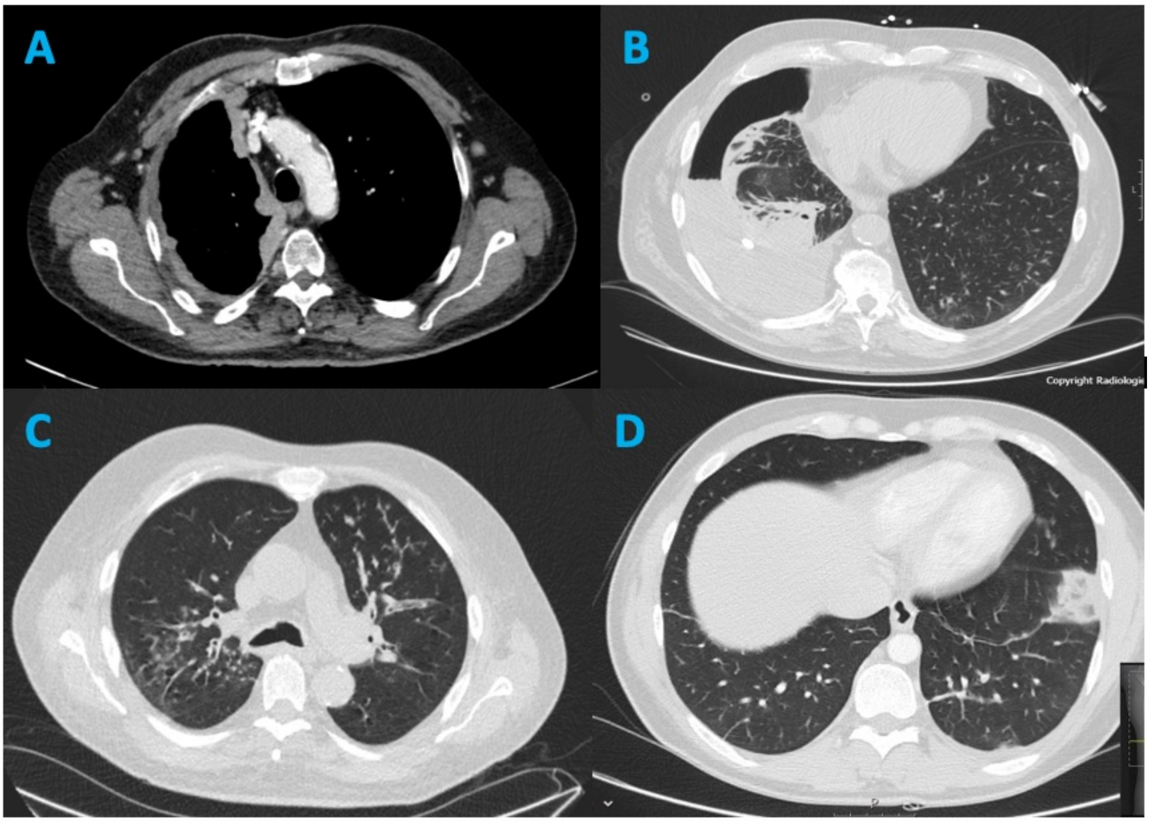
A
B
C
D
Copyright Radiologie

32. **Please assign the most fitting chest X-ray image (A-D) to the CT scan depicted below:**

- ◯ A
- ◯ B
- ◯ C
- ◯ D

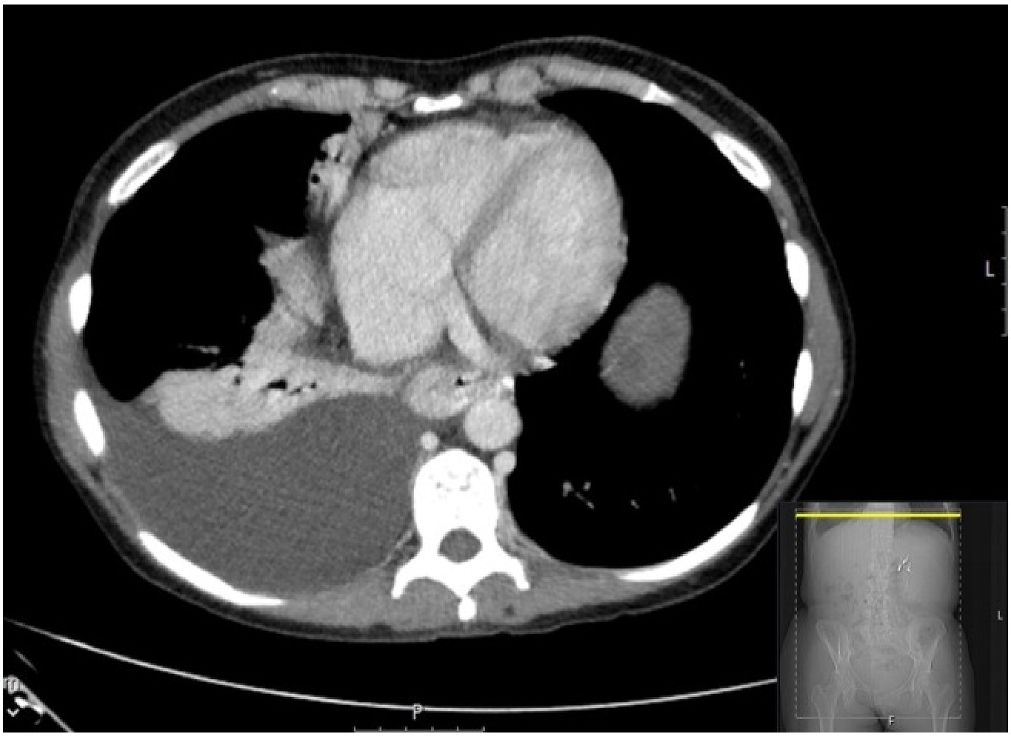

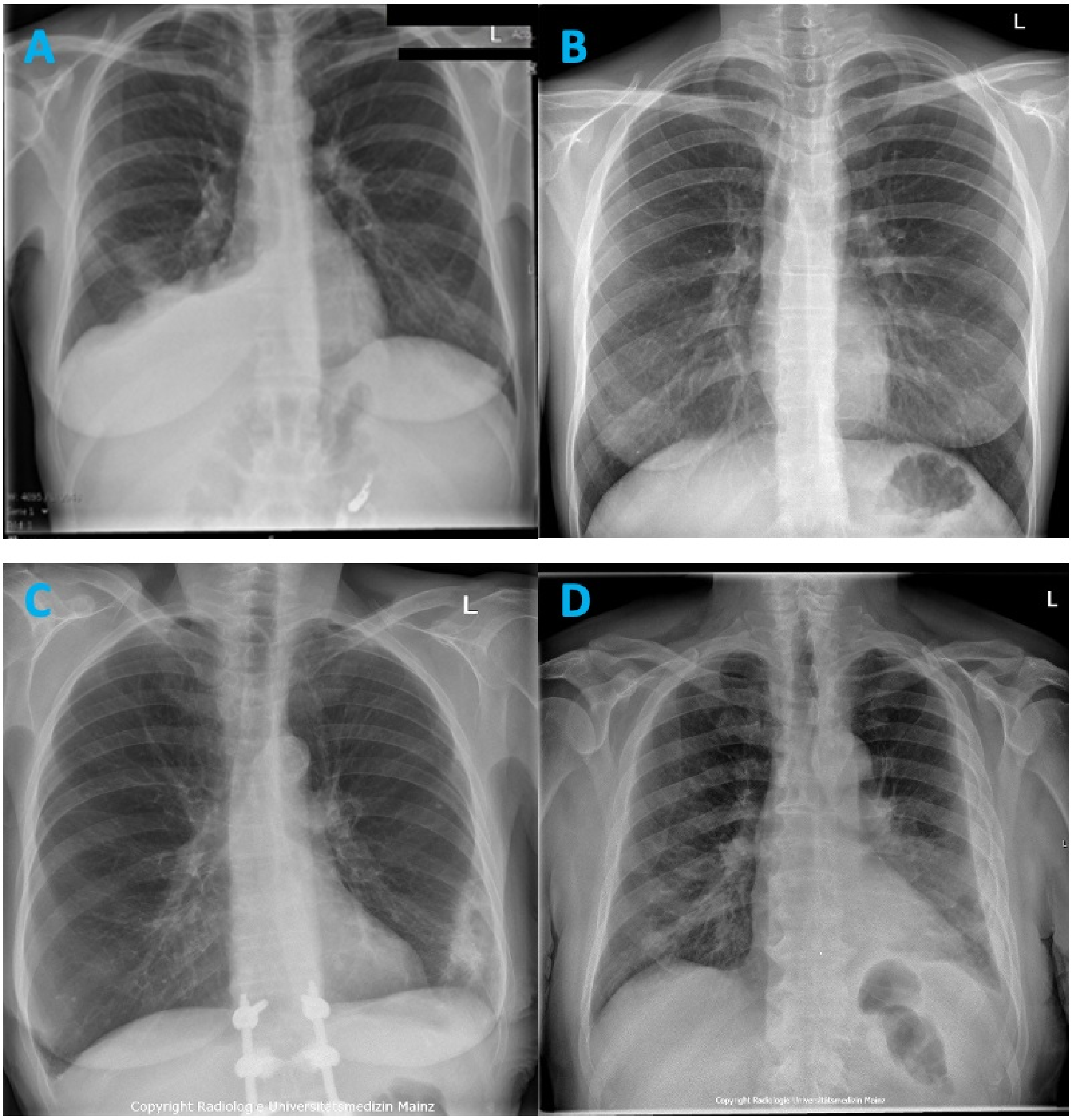

33. **Please assign the most fitting CT scan (A-D) to the chest X-ray depicted below:**

◯ A

◯ B

◯ C

◯ D

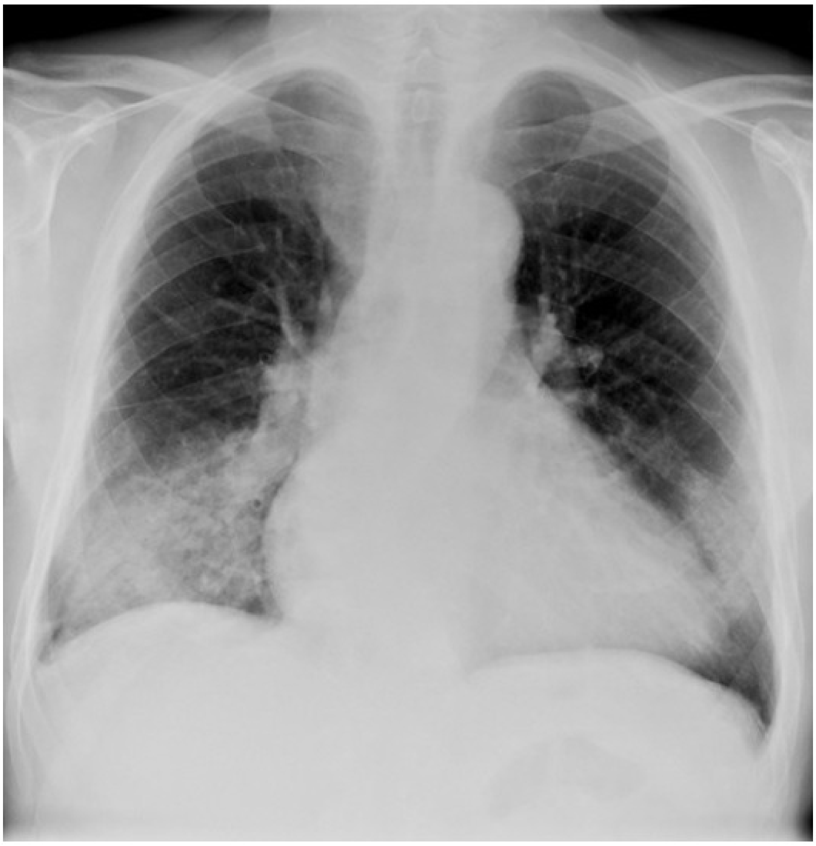

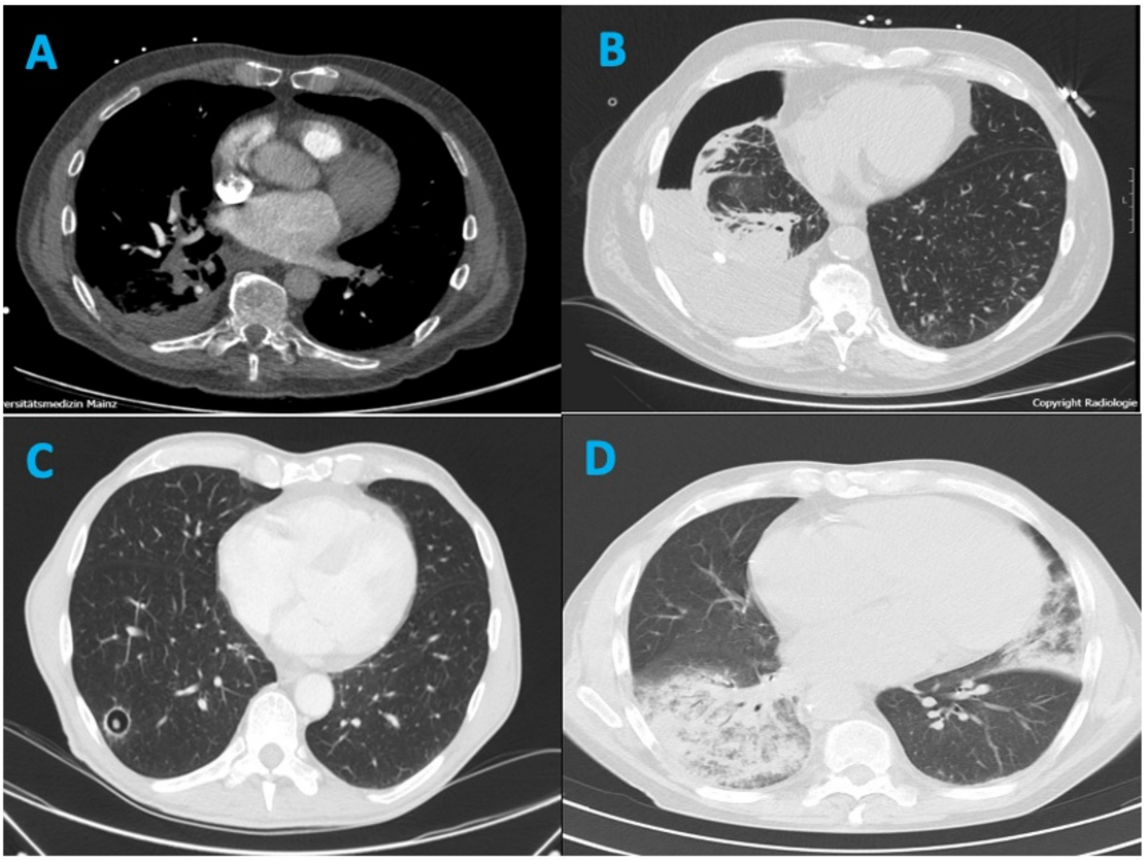
A
B
Copyright Radiologie
C
D

34. **Please assign the most fitting ultrasonogram (A-D) to the chest X-ray depicted below:**

- ◯ A
- ◯ B
- ◯ C
- ◯ D

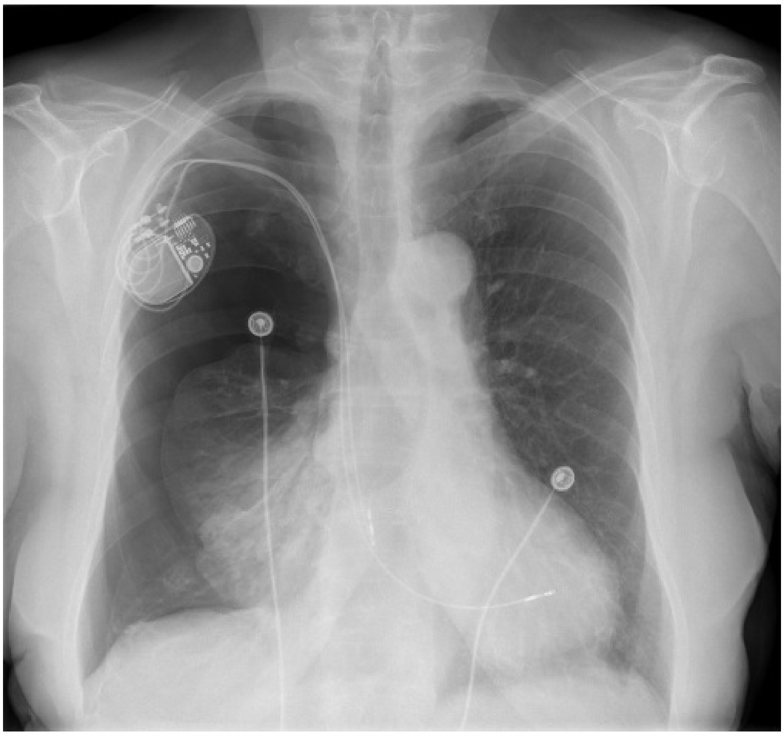

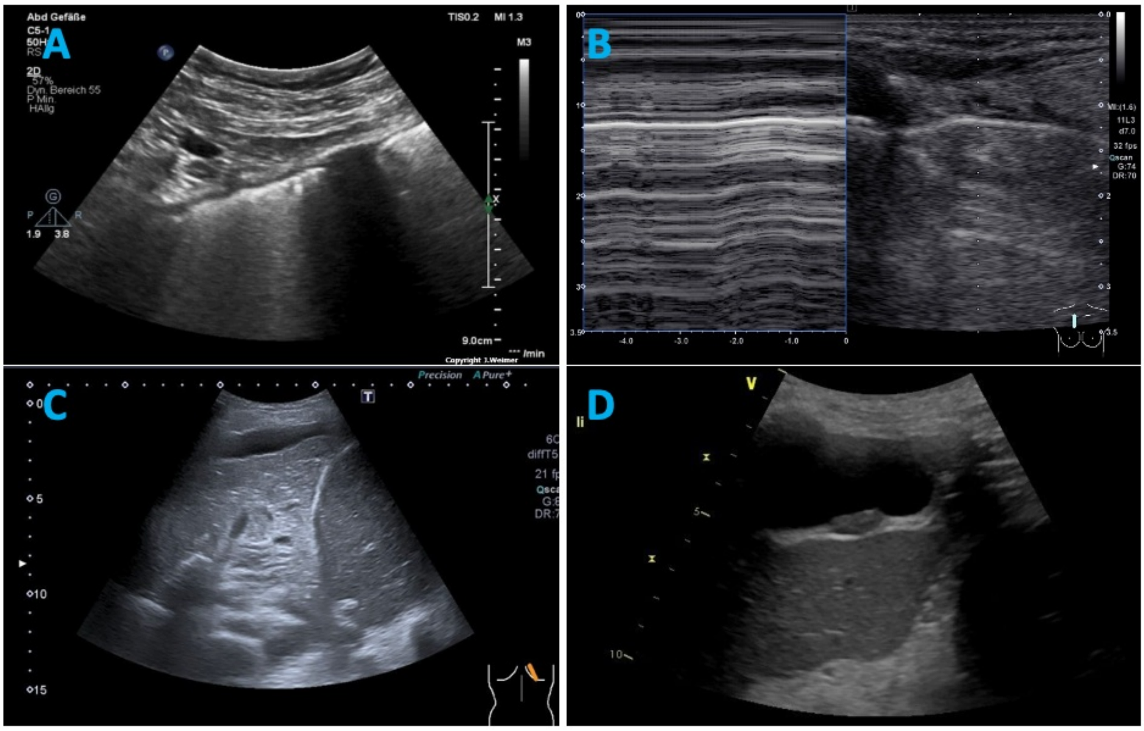
A
Abd Gefäße
TIS0.2 MI 1.3
9.0cm
Copyright J.Weimer
B
C
Precision
D
V
li
