# Supplementary material for: A blended learning approach for teaching thoracic radiology to medical students: a proof-of-concept study
Source: Front Med (Lausanne). 2023 Nov 23;10:1272893. doi: 10.3389/fmed.2023.1272893 (PMC10701891; doi:10.3389/fmed.2023.1272893)
Supplement: SUPPLEMENTARY TABLE S5 — Demographic characteristics of the participants. [file Data_Sheet_5.pdf]

Supplementary Table S5. Demographic characteristics of the participants.

Gender*	Female n (%)	93 (59.6%)	N/A n (%) 0 (0.0%)
	Male n (%)	63 (40.4%)	
Age	Mean y (SD)	25.7 (3.1)	N/A n (%) 0 (0.0%)
Study year	Mean y (SD)	4 (0.5)	N/A n (%) 0 (0.0%)
Medical training prior to medical university	"yes" n (%)	109 (70.0%)	N/A n (%) 0 (0.0%)
	"no" n (%)	47 (30.0%)	

SD=standard deviation, N/A=not available. *Answer options included "other" as well, which was not chosen by any participant.
