# Supplementary material for: A blended learning approach for teaching thoracic radiology to medical students: a proof-of-concept study
Source: Front Med (Lausanne). 2023 Nov 23;10:1272893. doi: 10.3389/fmed.2023.1272893 (PMC10701891; doi:10.3389/fmed.2023.1272893)
Supplement: INTERNAL QUALITY CONTROL S6 — Examination results from the year 2021 compared to the examination results from 2022 (the year in which our study took place) (“test”). [file Data_Sheet_6.pdf]

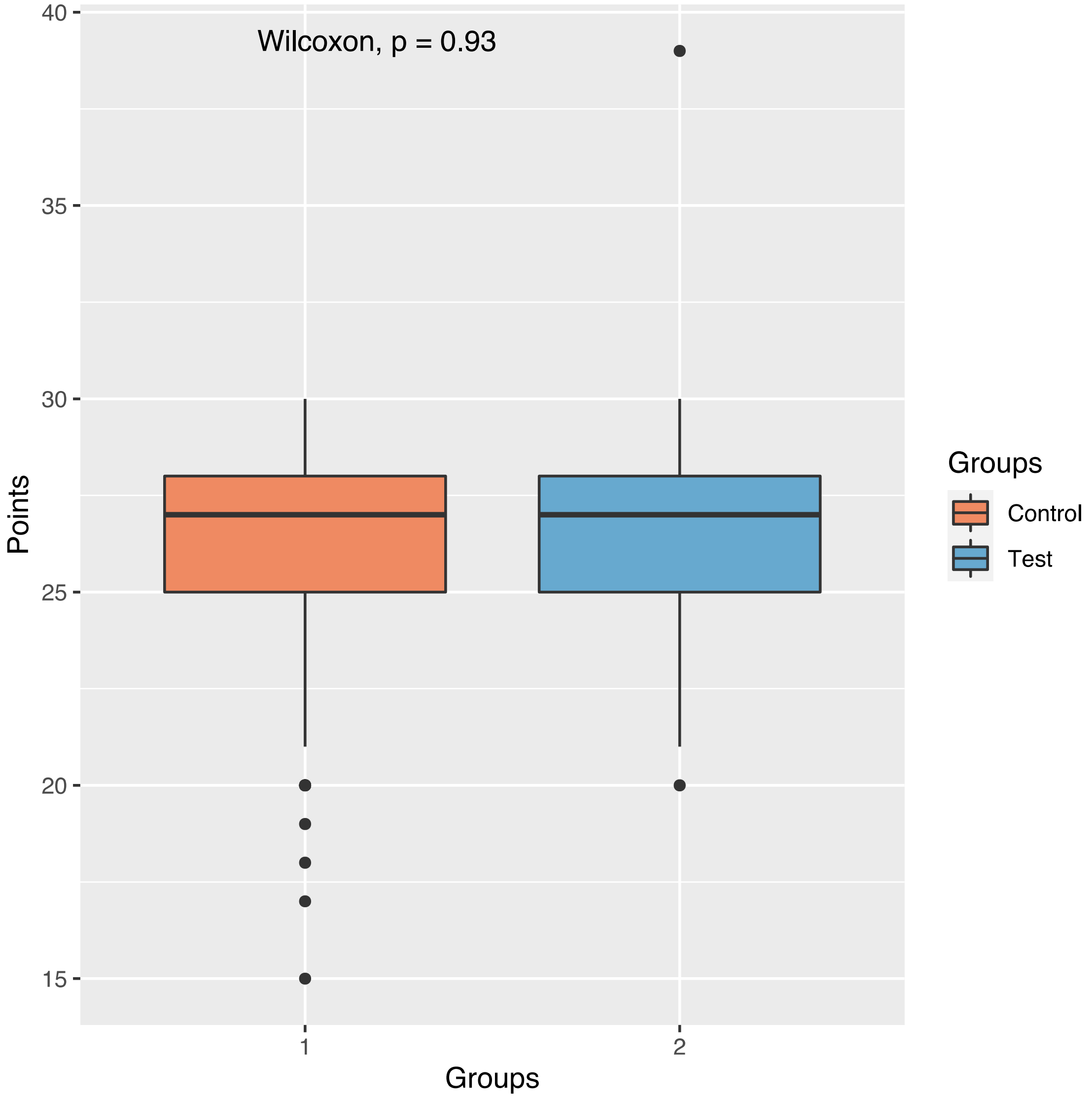

Internal quality control S6. Examination results from the year 2021 compared to the examination results from 2022 (the year in which our study took place) ("test").
