# Supplementary material for: A blended learning approach for teaching thoracic radiology to medical students: a proof-of-concept study
Source: Front Med (Lausanne). 2023 Nov 23;10:1272893. doi: 10.3389/fmed.2023.1272893 (PMC10701891; doi:10.3389/fmed.2023.1272893)
Supplement: SUPPLEMENTARY TABLE S7 — Completion rates of the modules (%). [file Data_Sheet_7.pdf]

Supplementary Table S7. Completion rates of the modules (%).

Online module	Completion (yes/no)	%
Basics chest X-ray	yes	80.6
	no	19.4
Basics chest CT	yes	78.2
	no	21.8
Basics ultrasonography	yes	71.8
	no	28.2
Pathology chest imaging	yes	68.5
	no	31.5
Pathology CT and X-ray	yes	66.9
	no	33.1
Pathology ultrasonography	yes	58.1
	no	41.9
No module	yes	11.2
	no	88.7
