# Supplementary material for: A blended learning approach for teaching thoracic radiology to medical students: a proof-of-concept study
Source: Front Med (Lausanne). 2023 Nov 23;10:1272893. doi: 10.3389/fmed.2023.1272893 (PMC10701891; doi:10.3389/fmed.2023.1272893)
Supplement: SUPPLEMENTARY TABLE S8 — Results of the regression analysis investigating various influencing factors regarding their impact on the objective knowledge test (“posttest”). [file Data_Sheet_8.pdf]

Supplementary Table S8. Results of the regression analysis investigating various influencing factors regarding their impact on the objective knowledge test (“posttest”).

„No module“			
**Item in the posttest**	**No module completed** (M±SD)	**Any module completed** (M±SD)	**p-value**
<u>Overall result</u> posttest	0.34±0.09	0.55±0.13	<0.001
<u>Overall result</u> „Basic modules“	0.47±0.09	0.73±0.17	<0.001
Result „Basics X-Ray“	0.32±0.32	0.71±0.31	<0.001
Result „Basics CT“	0.57±0.3	0.82±0.23	<0.001
Result „Basics US“	0.36±0.23	0.66±0.23	<0.001
<u>Overall result</u> „Patho modules“	0.22±0.1	0.38±0.14	<0.001
Result „Patho chest imaging“	0.51±0.2	0.65±0.22	0.03564
Result “Patho X-ray”	0.11±0.12	0.3±0.21	<0.001
Result „Patho CT“	0.09±0.13	0.23±0.18	<0.001
Result „Patho US“	0.09±0.16	0.28±0.26	<0.001
**Module „Basics X-ray“**			
**Item in the posttest**	**Completed** (M±SD	**Not completed** M±SD)	**p-value**
<u>Overall result</u> posttest	0.56±0.12	0.37±0.11	<0.001
<u>Overall result</u> „Basic modules“	0.75±0.15	0.5±0.15	<0.001
Result „Basics X-Ray“	0.74±0.3	0.38±0.3	<0.001
Result „Basics CT“	0.84±0.21	0.62±0.32	<0.001
Result „Basics US“	0.68±0.23	0.41±0.24	<0.001
<u>Overall result</u> „Patho modules“	0.39±0.14	0.24±0.1	<0.001
Result „Patho chest imaging“	0.66±0.23	0.53±0.19	0.01
Result “Patho X-ray”	0.31±0.21	0.13±0.17	<0.001

Result „Patho CT“	0.23±0.18	0.13±0.15	<0.001
Result „Patho US“	0.3±0.26	0.08±0.14	<0.001
**Module „Basics CT“**			
**Item in the posttest**	**Completed** (M±SD)	**Not completed** (M±SD)	**p-value**
Overall result posttest	0.56±0.13	0.4±0.13	<0.001
Overall result „Basic modules“	0.74±0.15	0.54±0.18	<0.001
Result „Basics X-Ray“	0.73±0.3	0.43±0.33	<0.001
Result „Basics CT“	0.84±0.21	0.62±0.32	<0.001
Result „Basics US“	0.67±0.23	0.46±0.26	<0.001
Overall result „Patho modules“	0.39±0.14	0.26±0.11	<0.001
Result „Patho chest imaging“	0.66±0.23	0.55±0.2	0.01919
Result “Patho X-ray”	0.31±0.21	0.16±0.18	<0.001
Result „Patho CT“	0.24±0.18	0.12±0.14	<0.001
Result „Patho US“	0.3±0.26	0.13±0.21	<0.001
**Module „Basics ultrasonography“**			
**Item in the posttest**	**Completed** (M±SD)	**Not completed** (M±SD)	**p-value**
Overall result posttest	0.57±0.12	0.4±0.12	<0.001
Overall result „Basic modules“	0.76±0.15	0.56±0.17	<0.001
Result „Basics X-Ray“	0.75±0.29	0.46±0.33	<0.001
Result „Basics CT“	0.83±0.23	0.7±0.28	<0.001
Result „Basics US“	0.71±0.21	0.42±0.23	<0.001
Overall result „Patho modules“	0.4±0.14	0.26±0.12	<0.001
Result „Patho chest imaging“	0.67±0.21	0.54±0.24	<0.001
Result “Patho X-ray”	0.32±0.21	0.16±0.18	<0.001
Result „Patho CT“	0.24±0.17	0.14±0.16	<0.001

Result „Patho US“	0.32±0.26	0.1±0.17	<0.001
**Module „Pathological findings chest imaging“**			
**Item in the posttest**	**Completed**<br>(M±SD)	**Not completed**<br>(M±SD)	**p-value**
Overall result<br>posttest	0.57±0.13	0.43±0.13	<0.001
Overall result<br>„Basic modules“	0.75±0.16	0.6±0.18	<0.001
Result „Basics X-Ray“	0.75±0.27	0.49±0.37	<0.001
Result „Basics CT“	0.84±0.23	0.7±0.28	<0.001
Result „Basics US“	0.68±0.22	0.49±0.26	<0.001
Overall result<br>„Patho modules“	0.4±0.15	0.28±0.11	<0.001
Result „Patho chest imaging“	0.67±0.21	0.54±0.22	0.00347
Result “Patho X-ray”	0.31±0.22	0.2±0.19	<0.001
Result „Patho CT“	0.25±0.18	0.13±0.14	<0.001
Result „Patho US“	0.31±0.26	0.15±0.21	<0.001
**Module „Pathological findings CT and X-ray“**			
**Item in the posttest**	**Completed**<br>(M±SD)	**Not completed**<br>(M±SD)	**p-value**
Overall result<br>posttest	0.58±0.12	0.42±0.13	<0.001
Overall result<br>„Basic modules“	0.76±0.14	0.58±0.18	<0.001
Result „Basics X-Ray“	0.77±0.27	0.46±0.34	<0.001
Result „Basics CT“	0.84±0.22	0.7±0.29	<0.001
Result „Basics US“	0.7±0.21	0.47±0.27	<0.001
Overall result<br>„Patho modules“	0.4±0.14	0.28±0.12	<0.001
Result „Patho chest imaging“	0.67±0.21	0.55±0.23	<0.001
Result “Patho X-ray”	0.31±0.22	0.21±0.19	<0.001
Result „Patho CT“	0.24±0.18	0.15±0.16	<0.001
Result „Patho US“	0.33±0.26	0.12±0.19	<0.001

Module „Pathological findings ultrasonography“			
**Item in the posttest**	**Completed**<br>(M±SD)	**Not completed**<br>(M±SD)	**p-value**
Overall result<br>posttest	0.59±0.12	0.43±0.12	<0.001
Overall result<br>„Basic modules“	0.77±0.15	0.61±0.18	<0.001
Result „Basics X-Ray“	0.75±0.28	0.55±0.36	<0.001
Result „Basics CT“	0.86±0.21	0.7±0.27	<0.001
Result „Basics US“	0.72±0.2	0.49±0.26	<0.001
Overall result<br>„Patho modules“	0.43±0.13	0.27±0.12	<0.001
Result „Patho chest imaging“	0.71±0.17	0.52±0.25	<0.001
Result “Patho X-ray”	0.33±0.22	0.19±0.18	<0.001
Result „Patho CT“	0.26±0.17	0.14±0.16	<0.001
Result „Patho US“	0.35±0.26	0.13±0.2	<0.001
