# Supplementary material for: A blended learning approach for teaching thoracic radiology to medical students: a proof-of-concept study
Source: Front Med (Lausanne). 2023 Nov 23;10:1272893. doi: 10.3389/fmed.2023.1272893 (PMC10701891; doi:10.3389/fmed.2023.1272893)
Supplement: SUPPLEMENTARY TABLE S9 — Results of the regression analysis investigating various influencing factors regarding their impact on the objective knowledge test (“pretest”). [file Data_Sheet_9.pdf]

Supplementary Table S9. Results of the regression analysis investigating various influencing factors regarding their impact on the objective knowledge test ("pretest").

**Gender**			
**Item in the pretest**	**male** (MW±SD)	**female** (MW±SD)	**p-value**
Overall result posttest	0.32±0.12	0.33±0.13	0.8396
Overall result „Basic modules"	0.44±0.16	0.42±0.16	0.3846
Result „Basics X-Ray"	0.3±0.31	0.3±0.35	0.9221
Result „Basics CT"	0.47±0.3	0.33±0.31	0.01286
Result „Basics US"	0.47±0.19	0.43±0.24	0.3559
Overall result „Patho modules"	0.21±0.12	0.24±0.13	0.1331
Result „Patho chest imaging"	0.37±0.23	0.43±0.24	0.169
Result "Patho X-ray"	0.17±0.15	0.19±0.15	0.439
Result „Patho CT"	0.15±0.16	0.15±0.16	0.8114
Result „Patho US"	0.06±0.12	0.12±0.18	0.02542

**Prior professional experience**			
**Item in the pretest**	**yes** (MW±SD)	**no** (MW±SD)	**p-value**
Overall result posttest	0.33±0.13	0.31±0.09	0.3851
Overall result „Basic modules"	0.44±0.17	0.4±0.14	0.1661
Result „Basics X-Ray"	0.31±0.34	0.26±0.33	0.4921
Result „Basics CT"	0.4±0.31	0.31±0.3	0.1729
Result „Basics US"	0.46±0.23	0.42±0.18	0.2939
Overall result „Patho modules"	0.23±0.13	0.23±0.1	0.8887
Result „Patho chest imaging"	0.4±0.25	0.44±0.23	0.4273
Result "Patho X-ray"	0.18±0.16	0.17±0.13	0.7128
Result „Patho CT"	0.16±0.17	0.15±0.14	0.7532
Result „Patho US"	0.11±0.17	0.09±0.16	0.5985

**Prior experience in imaging**			
**Item in the pretest**	**yes** (MW±SD)	**no** (MW±SD)	**p-value**

Overall result posttest	0.39±0.14	0.3±0.14	<0.001
Overall result „Basic modules“	0.51±0.19	0.4±0.14	<0.001
Result „Basics X-Ray“	0.44±0.34	0.24±0.32	<0.001
Result „Basics CT“	0.47±0.34	0.34±0.29	0.05315
Result „Basics US“	0.54±0.22	0.41±0.21	<0.001
Overall result „Patho modules“	0.29±0.12	0.21±0.12	<0.001
Result „Patho chest imaging“	0.47±0.25	0.38±0.24	0.08372
Result “Patho X-ray”	0.24±0.16	0.16±0.15	0.009425
Result „Patho CT“	0.21±0.18	0.13±0.15	0.03192
Result „Patho US“	0.14±0.19	0.09±0.15	0.1446
**Prior use of learning resources for thoracic radiology**			
**Item in the pretest**	**yes** (MW±SD)	**no** MW±SD	**p-value**
Overall result posttest	0.35±0.14	0.32±0.12	0.2993
Overall result „Basic modules“	0.47±0.18	0.42±0.16	0.1693
Result „Basics X-Ray“	0.43±0.32	0.27±0.34	0.0402
Result „Basics CT“	0.47±0.32	0.35±0.31	0.1373
Result „Basics US“	0.5±0.24	0.44±0.21	0.2745
Overall result „Patho modules“	0.24±0.13	0.23±0.12	0.7307
Result „Patho chest imaging“	0.41±0.23	0.41±0.25	0.9817
Result “Patho X-ray”	0.19±0.16	0.18±0.15	0.7475
Result „Patho CT“	0.18±0.18	0.15±0.16	0.4662
Result „Patho US“	0.09±0.17	0.1±0.16	0.6432

T2

Gender			
**Item in the posttest**	**male** (MW±SD)	**female** (MW±SD)	**p-value**
Overall result posttest	0.5±0.14	0.54±0.14	0.2545
Overall result „Basic modules“	0.7±0.18	0.7±0.18	0.8775
Result „Basics X-Ray“	0.63±0.36	0.69±0.31	0.3831
Result „Basics CT“	0.76±0.26	0.81±0.25	0.2746
Result „Basics US“	0.63±0.22	0.62±0.27	0.7563
Overall result „Patho modules“	0.32±0.32	0.38±0.15	0.04617
Result „Patho chest imaging“	0.6±0.25	0.65±0.21	0.2118
Result “Patho X-ray”	0.25±0.21	0.29±0.22	0.4079
Result „Patho CT“	0.17±0.17	0.24±0.18	0.02941
Result „Patho US“	0.22±0.24	0.28±0.26	0.1723

Prior professional experience			
**Item in the posttest**	**yes** (MW±SD)	**no** (MW±SD)	**p-value**
Overall result posttest	0.52±0.15	0.53±0.13	0.6985
Overall result „Basic modules“	0.69±0.19	0.73±0.16	0.247
Result „Basics X-Ray“	0.67±0.34	0.66±0.29	0.9373
Result „Basics CT“	0.77±0.26	0.85±0.22	0.09115
Result „Basics US“	0.62±0.25	0.62±0.24	0.9781
Overall result „Patho modules“	0.36±0.16	0.35±0.12	0.5319
Result „Patho chest imaging“	0.64±0.24	0.61±0.19	0.3836
Result “Patho X-ray”	0.28±0.22	0.27±0.18	0.9589
Result „Patho CT“	0.22±0.18	0.2±0.17	0.6193
Result „Patho US“	0.26±0.26	0.26±0.26	0.9424

Prior experience in imaging			
**Item in the posttest**	**yes** (MW±SD)	**no** (MW±SD)	**p-value**

Overall result posttest	0.58±0.14	0.5±0.14	0.01233
Overall result „Basic modules“	0.76±0.18	0.68±0.18	0.02532
Result „Basics X-Ray“	0.72±0.35	0.64±0.32	0.276
Result „Basics CT“	0.83±0.22	0.78±0.26	0.2559
Result „Basics US“	0.72±0.22	0.59±0.25	0.00429
Overall result „Patho modules“	0.41±0.15	0.34±0.14	0.02752
Result „Patho chest imaging“	0.66±0.26	0.62±0.21	0.4692
Result “Patho X-ray”	0.33±0.23	0.25±0.2	0.08225
Result „Patho CT“	0.26±0.16	0.2±0.18	0.0666
Result „Patho US“	0.35±0.28	0.23±0.24	0.03554

**Prior use of learning resources for thoracic radiology**			
**Item in the posttest**	**yes** (MW±SD)	**no** (MW±SD)	**p-value**
Overall result posttest	0.55±0.13	0.52±0.15	0.2684
Overall result „Basic modules“	0.74±0.15	0.69±0.18	0.2189
Result „Basics X-Ray“	0.7±0.33	0.66±0.33	0.5463
Result „Basics CT“	0.84±0.17	0.78±0.27	0.2066
Result „Basics US“	0.64±0.24	0.62±0.25	0.7159
Overall result „Patho modules“	0.38±0.14	0.36±0.15	0.4524
Result „Patho chest imaging“	0.72±0.17	0.61±0.23	0.01976
Result “Patho X-ray”	0.29±0.2	0.27±0.22	0.7317
Result „Patho CT“	0.19±0.19	0.22±0.18	0.4699
Result „Patho US“	0.26±0.26	0.26±0.26	0.9301

**„No module“**			
**Item in the posttest**	**No module completed** (MW±SD)	**Any module completed** (MW±SD)	**p-value**
Overall result posttest	0.34±0.09	0.55±0.13	<0.001
Overall result „Basic modules“	0.47±0.09	0.73±0.17	<0.001
Result „Basics X-Ray“	0.32±0.32	0.71±0.31	<0.001
Result „Basics CT“	0.57±0.3	0.82±0.23	<0.001
Result „Basics US“	0.36±0.23	0.66±0.23	<0.001
Overall result	0.22±0.1	0.38±0.14	<0.001

„Patho modules“			
Result „Patho chest imaging“	0.51±0.2	0.65±0.22	0.03564
Result “Patho X-ray”	0.11±0.12	0.3±0.21	<0.001
Result „Patho CT“	0.09±0.13	0.23±0.18	<0.001
Result „Patho US“	0.09±0.16	0.28±0.26	<0.001
**Module „Basics X-ray“**			
**Item in the posttest**	**Completed** (MW±SD	**Not completed** MW±SD)	**p-value**
Overall result posttest	0.56±0.12	0.37±0.11	<0.001
Overall result „Basic modules“	0.75±0.15	0.5±0.15	<0.001
Result „Basics X-Ray“	0.74±0.3	0.38±0.3	<0.001
Result „Basics CT“	0.84±0.21	0.62±0.32	<0.001
Result „Basics US“	0.68±0.23	0.41±0.24	<0.001
Overall result „Patho modules“	0.39±0.14	0.24±0.1	<0.001
Result „Patho chest imaging“	0.66±0.23	0.53±0.19	0.01
Result “Patho X-ray”	0.31±0.21	0.13±0.17	<0.001
Result „Patho CT“	0.23±0.18	0.13±0.15	<0.001
Result „Patho US“	0.3±0.26	0.08±0.14	<0.001
**Module „Basics CT“**			
**Item in the posttest**	**Completed** (MW±SD)	**Not completed** (MW±SD)	**p-value**
Overall result posttest	0.56±0.13	0.4±0.13	<0.001
Overall result „Basic modules“	0.74±0.15	0.54±0.18	<0.001
Result „Basics X-Ray“	0.73±0.3	0.43±0.33	<0.001
Result „Basics CT“	0.84±0.21	0.62±0.32	<0.001
Result „Basics US“	0.67±0.23	0.46±0.26	<0.001
Overall result „Patho modules“	0.39±0.14	0.26±0.11	<0.001
Result „Patho chest imaging“	0.66±0.23	0.55±0.2	0.01919
Result “Patho X-ray”	0.31±0.21	0.16±0.18	<0.001
Result „Patho CT“	0.24±0.18	0.12±0.14	<0.001
Result „Patho US“	0.3±0.26	0.13±0.21	<0.001
**Module „Basics ultrasonography“**			
**Item in the posttest**	**Completed** (MW±SD)	**Not completed** (MW±SD)	**p-value**
Overall result posttest	0.57±0.12	0.4±0.12	<0.001
Overall result	0.76±0.15	0.56±0.17	<0.001

„Basic modules“			
Result „Basics X-Ray“	0.75±0.29	0.46±0.33	<0.001
Result „Basics CT“	0.83±0.23	0.7±0.28	<0.001
Result „Basics US“	0.71±0.21	0.42±0.23	<0.001
Overall result „Patho modules“	0.4±0.14	0.26±0.12	<0.001
Result „Patho chest imaging“	0.67±0.21	0.54±0.24	<0.001
Result “Patho X-ray”	0.32±0.21	0.16±0.18	<0.001
Result „Patho CT“	0.24±0.17	0.14±0.16	<0.001
Result „Patho US“	0.32±0.26	0.1±0.17	<0.001
**Module „Pathological findings chest imaging“**			
**Item in the posttest**	**Completed** (MW±SD)	**Not completed** (MW±SD)	**p-value**
Overall result posttest	0.57±0.13	0.43±0.13	<0.001
Overall result „Basic modules“	0.75±0.16	0.6±0.18	<0.001
Result „Basics X-Ray“	0.75±0.27	0.49±0.37	<0.001
Result „Basics CT“	0.84±0.23	0.7±0.28	<0.001
Result „Basics US“	0.68±0.22	0.49±0.26	<0.001
Overall result „Patho modules“	0.4±0.15	0.28±0.11	<0.001
Result „Patho chest imaging“	0.67±0.21	0.54±0.22	0.00347
Result “Patho X-ray”	0.31±0.22	0.2±0.19	<0.001
Result „Patho CT“	0.25±0.18	0.13±0.14	<0.001
Result „Patho US“	0.31±0.26	0.15±0.21	<0.001
**Module „Pathological findings CT and X-ray“**			
**Item in the posttest**	**Completed** (MW±SD)	**Not completed** (MW±SD)	**p-value**
Overall result posttest	0.58±0.12	0.42±0.13	<0.001
Overall result „Basic modules“	0.76±0.14	0.58±0.18	<0.001
Result „Basics X-Ray“	0.77±0.27	0.46±0.34	<0.001
Result „Basics CT“	0.84±0.22	0.7±0.29	<0.001
Result „Basics US“	0.7±0.21	0.47±0.27	<0.001
Overall result „Patho modules“	0.4±0.14	0.28±0.12	<0.001
Result „Patho chest imaging“	0.67±0.21	0.55±0.23	<0.001
Result “Patho X-ray”	0.31±0.22	0.21±0.19	<0.001
Result „Patho CT“	0.24±0.18	0.15±0.16	<0.001
Result „Patho US“	0.33±0.26	0.12±0.19	<0.001
**Module „Pathological findings ultrasonography“**			

**Item in the posttest**	**Completed** (MW±SD)	**Not completed** (MW±SD)	**p-value**
Overall result posttest	0.59±0.12	0.43±0.12	<0.001
Overall result „Basic modules“	0.77±0.15	0.61±0.18	<0.001
Result „Basics X-Ray“	0.75±0.28	0.55±0.36	<0.001
Result „Basics CT“	0.86±0.21	0.7±0.27	<0.001
Result „Basics US“	0.72±0.2	0.49±0.26	<0.001
Overall result „Patho modules“	0.43±0.13	0.27±0.12	<0.001
Result „Patho chest imaging“	0.71±0.17	0.52±0.25	<0.001
Result “Patho X-ray”	0.33±0.22	0.19±0.18	<0.001
Result „Patho CT“	0.26±0.17	0.14±0.16	<0.001
Result „Patho US“	0.35±0.26	0.13±0.2	<0.001
