# Supplementary material for: A blended learning approach for teaching thoracic radiology to medical students: a proof-of-concept study
Source: Front Med (Lausanne). 2023 Nov 23;10:1272893. doi: 10.3389/fmed.2023.1272893 (PMC10701891; doi:10.3389/fmed.2023.1272893)
Supplement: SUPPLEMENTARY TABLE S10 — (Sub-)results of the knowledge tests, broken down by each module separately. [file Data_Sheet_10.PDF]

Table S10: (Sub-)results of the knowledge tests, broken down by each module separately. Results (in %) in the pre- and posttest (mean ± standard deviation) and differences including statistical significances (p-value) and effect size (Cohen's d) are presented.

**Item**	**Pretest** (M±SD in %)	**Posttest** (M±SD in %)	**Difference** (M±SD in %)	**p-value**	**Effect size** (Cohen's d)
<u>Overall result</u> "Basics modules"	0.43±0.16	0.7±0.18	0.27±0.18	<0.001	1.60
Result „Basics X-Ray"	0.3±0.34	0.67±0.33	0.37±0.39	<0.001	1.10
Result „Basics CT"	0.38±0.32	0.79±0.25	0.42±0.36	<0.001	1.48
Result „Basics ultrasonography"	0.45±0.22	0.62±0.25	0.18±0.23	<0.001	0.75
<u>Overall result</u> "Patho modules"	0.23±0.12	0.36±0.15	0.13±0.14	<0.001	0.96
Result „Patho chest imaging"	0.41±0.24	0.63±0.22	0.22±0.30	<0.001	0.96
Result „Patho X-Ray"	0.18±0.15	0.28±0.21	0.09±0.22	<0.001	0.51
Result „Patho CT"	0.15±0.16	0.21±0.18	0.06±0.20	<0.001	0.36
Result „Patho ultrasonography"	0.1±0.16	0.26±0.26	0.16±0.24	<0.001	0.74
