# Supplementary material for: A blended learning approach for teaching thoracic radiology to medical students: a proof-of-concept study
Source: Front Med (Lausanne). 2023 Nov 23;10:1272893. doi: 10.3389/fmed.2023.1272893 (PMC10701891; doi:10.3389/fmed.2023.1272893)
Supplement: SUPPLEMENTARY FIGURE S2 — Examples of several features which were included to enrich the teaching content of the online learning platform. [file Image_2.pdf]

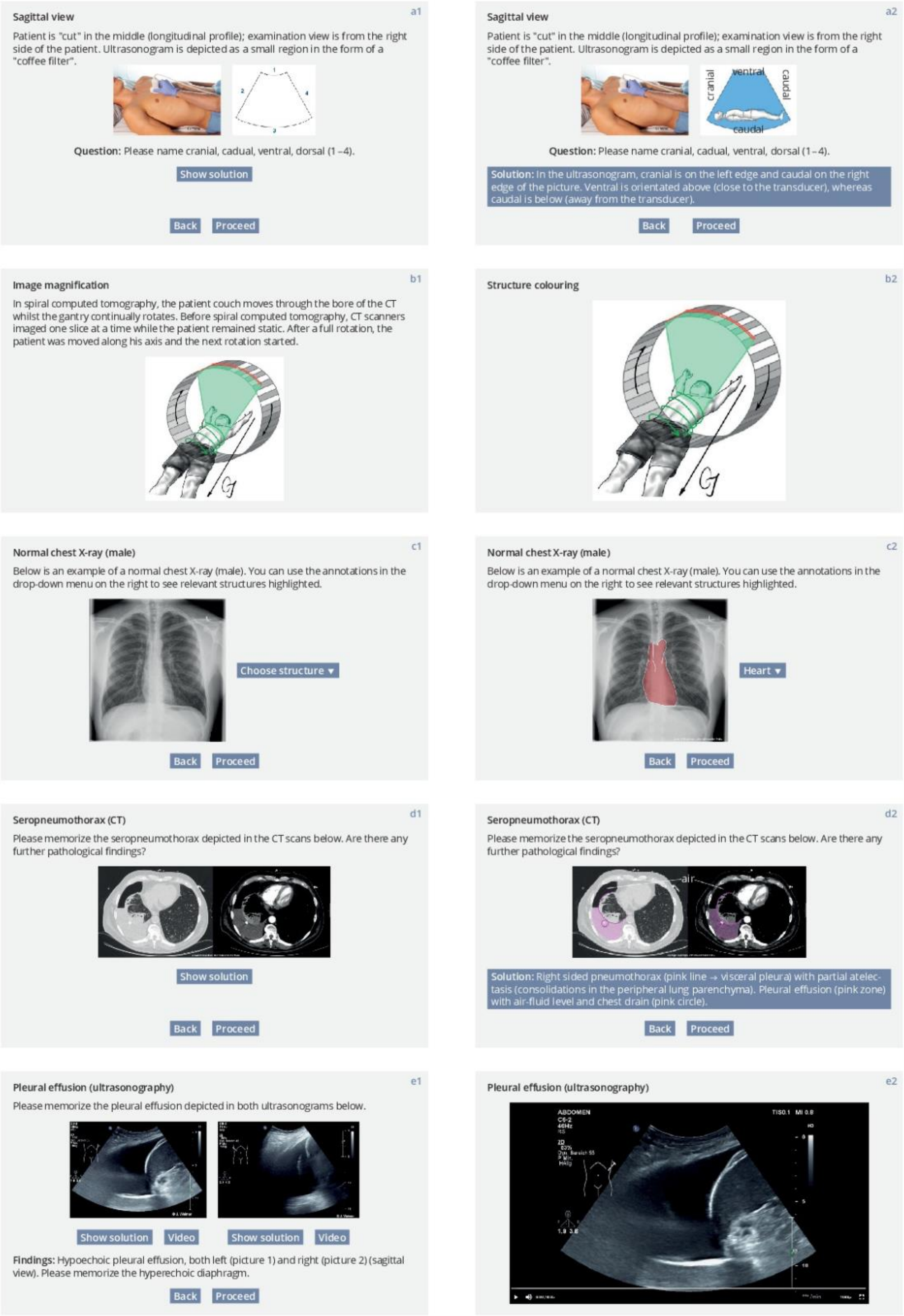

Supplementary Figure S2. Examples of several features which were included to enrich the teaching content of the online learning platform.
